# Diverse cell-specific patterns of alternative polyadenylation in *Drosophila*

**DOI:** 10.1038/s41467-022-32305-0

**Published:** 2022-09-13

**Authors:** Seungjae Lee, Yen-Chung Chen, FCA Consortium, Austin E. Gillen, J. Matthew Taliaferro, Bart Deplancke, Hongjie Li, Eric C. Lai

**Affiliations:** 1 Developmental Biology Program, Sloan Kettering Institute, 1275 York Ave, Box 252, New York, NY 10065 USA; 2 Department of Biology, New York University, New York, NY 10013 USA; 3 Division of Hematology, University of Colorado Anschutz Medical Campus, Aurora, CO USA; 4 Rocky Mountain Regional VA Medical Center, Aurora, CO USA; 5 RNA Bioscience Initiative, University of Colorado Anschutz Medical Campus, Aurora, CO USA; 6 Department of Biochemistry and Molecular Genetics, University of Colorado Anschutz Medical Campus, Aurora, CO USA; 7 Laboratory of Systems Biology and Genetics, Institute of Bio-engineering & Global Health Institute, School of Life Sciences, EPFL, CH-1015 Lausanne, Switzerland; 8 Huffington Center on Aging, Baylor College of Medicine, Houston, TX 77030 USA; 9 Department of Molecular and Human Genetics, Baylor College of Medicine, Houston, TX 77030 USA

**Keywords:** Gene regulatory networks, RNA splicing

## Abstract

Most genes in higher eukaryotes express isoforms with distinct 3’ untranslated regions (3’ UTRs), generated by alternative polyadenylation (APA). Since 3’ UTRs are predominant locations of post-transcriptional regulation, APA can render such programs conditional, and can also alter protein sequences via alternative last exon (ALE) isoforms. We previously used 3’-sequencing from diverse *Drosophila* samples to define multiple tissue-specific APA landscapes. Here, we exploit comprehensive single nucleus RNA-sequencing data (Fly Cell Atlas) to elucidate cell-type expression of 3’ UTRs across >250 adult *Drosophila* cell types. We reveal the cellular bases of multiple tissue-specific APA/ALE programs, such as 3’ UTR lengthening in differentiated neurons and 3’ UTR shortening in spermatocytes and spermatids. We trace dynamic 3’ UTR patterns across cell lineages, including in the male germline, and discover new APA patterns in the intestinal stem cell lineage. Finally, we correlate expression of RNA binding proteins (RBPs), miRNAs and global levels of cleavage and polyadenylation (CPA) factors in several cell types that exhibit characteristic APA landscapes, yielding candidate regulators of transcriptome complexity. These analyses provide a comprehensive foundation for future investigations of mechanisms and biological impacts of alternative 3’ isoforms across the major cell types of this widely-studied model organism.

## Abstract (web summary)

Single cell data provides cellular resolution on gene expression, but is rarely mined for isoforms. Analysis of 3' isoforms across ~250 Drosophila cell types reveals the cellular bases for numerous tissue-specific 3' programs, identifies new 3' programs, and nominates candidate trans-acting factors

## Introduction

It is common practice to refer colloquially to “the gene model”, a term that implies a single isoform is generated following transcription and processing of a given locus. In reality, the actions of alternative promoter selection, alternative exon choices, and alternative cleavage and polyadenylation sites – often in combinatorial fashion – can yield a dizzying array of distinct isoforms from an individual gene. As such, transcript isoforms can be subject to distinct regulatory regimes and/or encode distinct proteins. The biogenesis of transcript isoforms is regulated by diverse cis-motifs and trans-acting factors. Ongoing challenges of many current investigations are to understand the mechanistic bases of isoform generation, and the biological importance of generating multiple isoforms from a given locus.

Of these strategies that diversify isoforms, the breadth of alternative polyadenylation (APA) sites was recognized in the past decade. Once considered as oddities of mRNA processing^1–3^, the advent of deep sequencing made it abundantly clear that a majority of metazoan genes express multiple 3’ UTR isoforms^4,5^. Moreover, the relative abundance of 3’ UTR isoforms is neither fixed nor haphazard. Instead, genomic analyses have documented myriad APA dynamics that coordinately affect substantial cohorts of genes across development, organogenesis and cell types, e.g. in invertebrates^6–9^ and vertebrates^10–14^. As well, there are numerous instances where APA is globally altered according to cellular behavior and external stimuli^15–18^. Collectively, such studies implicate the existence of discrete regulatory mechanisms that can adjust the efficacy of cleavage and polyadenylation at particular sites during primary transcript processing, and/or post-transcriptional mechanisms that selectively stabilize or destabilize specific isoforms to bias the final pool of isoforms.

Although APA isoforms can be recognized and quantified from conventional RNA-seq data^19–21^, most RNA-seq protocols tend to undersample data at transcript termini (both 5’ and 3’). Instead, nucleotide-resolution of the positions of transcript polyadenylation can be obtained by utilizing some form of an oligo-dT primer to enhance priming near polyadenylation sites^4,5^. These “3’-seq” strategies, which collectively encompass a number of variant protocols^6,22–25^, have been the major means by which genomewide alterations in APA have been studied.

A limitation of current 3’-seq methods is their reliance on substantial quantities of RNA for library preparation. This has limited most atlases of “cell-specific” APA to reflect cells that can be grown in culture (e.g. immortalized cell lines or certain stem cells). Otherwise, most 3’-seq profilings of in vivo materials comprise whole animals or dissected tissues, which inevitably comprise many constituent cell types. Thus, for many tissue-specific APA trends, there is often only correlational evidence for the underlying cell type affected. For example, we characterized widespread neural 3’ UTR lengthening and testis 3’ UTR shortening in *Drosophila*^8,9,26^, but the underlying cell types affected were not directly assayed using genomic techniques. A small subset of 3’ UTR isoforms were tested using in situ hybridization^8,26^, which can provide cellular resolution. However, while this technique has utility to resolve cell-restricted 3’ UTR elongation, it does not distinguish shorter isoforms that might be coexpressed with longer isoforms.

The recent advent of single cell profiling techniques has radically upgraded possibilities to detect and quantify cell-specific chromatin features, transcripts, and even proteins. Of these, the techniques for single cell RNA-sequencing (scRNA-seq) are currently the most advanced and widely used. Although scRNA-seq platforms have been around now for several years and are broadly available in core facilities, outsourced services, and a growing number of individual labs, the vast majority of publications utilize single cell transcriptome data solely to classify marker gene expression to distinguish cell types. That is, scRNA-seq reads are collapsed into single values per gene per cell, and isoforms are not distinguished in downstream analyses. There is a practical consideration for this, as the sparse nature of single cell measurements hinder accurate assessments of gene expression, especially when attempting to quantify alternative isoforms^27,28^. Nevertheless, the availability of methods that permit scRNA-seq profiling across transcript bodies (e.g. Smart-seq2^29^), has encouraged single cell isoform analysis^30–33^. However, the majority of current scRNA-seq datasets do not sample across transcripts, but instead comprise 3’-biased sequence tags (e.g., 10X Genomics platform), conceptually similar to previous 3’-digital gene expression (3’-DGE) approaches^34,35^. While this does not permit assessment of 5’ or internal isoform variants, in principle this may permit interrogation of 3’ isoforms. Indeed, several mammalian-focused studies recently illustrated how scRNA-seq data can be applied to study APA at cellular resolution^36–38^.

We utilized the powerful *Drosophila* system to dissect APA landscapes^9^, mechanisms^39,40^ and biology^41,42^. Accordingly, we were motivated to address whether scRNA-seq data could elucidate cell-specific isoforms and programs. Although the number of *Drosophila* scRNA-seq studies is modest compared to those in mammals, they are growing. One major focus of fly single cell studies has been on characterizing neuronal diversity^43–47^, with additional studies addressing specific developmental stages and/or dissected tissues^48^. Clearly, though, scRNA-seq is rapidly becoming a more standard technology in *Drosophila*, as in mammals. In order to provide a unified and broad basis of assigning *Drosophila* cell types, we recently systematically characterized hundreds of distinct adult cell types across constituent tissues^49^. The efforts of the Fly Cell Atlas (FCA) consortium (https://flycellatlas.org/) provide a strong foundation to investigate 3’ isoforms in individual *Drosophila* cell types.

**Fig. 1:**
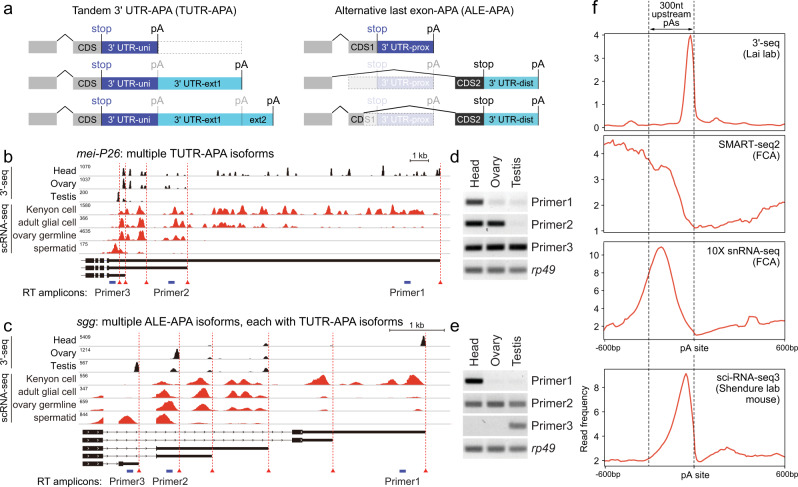
10X sNuc-seq data reveals cell-specific programs of 3’ isoforms. **a** Two classes of 3’ mRNA isoforms generated by alternative polyadenylation (APA). Left, tandem 3’ UTR (TUTR) isoforms encode the same open reading frame, but express different 3’ UTRs. Here, a gene model incorporates three different TUTR isoforms. Right, alternative last exon (ALE) isoforms harbor different C-terminal coding potential, in addition to non-overlapping 3’ UTR contents. While ALE isoforms could be generated as a result of splicing regulation, in at least some cases, ALE isoforms are determined via APA. Here, two examples of exon layouts that can generate distal ALE switching are shown. **b** Exemplar gene (*mei-P26*) with highly alternative TUTR isoforms evidence in bulk 3’-sequencing from the indicated tissues (black tracks), for which corresponding varieties of TUTR isoforms can be found in individual cell types originating from the same tissues (red tracks). **c** Exemplar gene (*sgg*) with diverse ALE isoforms (three distinct coding potentials), of which several of these are also subject to TUTR isoform generation; these collectively generate many different 3’ UTRs. Again, individual cell types from each of these tissues recapitulate the ALE and TUTR diversity seen in bulk 3’-seq data. **d**, **e** Semi-quantitative RT-PCR validation for tissue-specific expression of different 3’ UTR isoforms of *mei-P26* (**d**) and *sgg* (**e**). Data is representative of 2 independent experiments; amplicons are 100–200 bp. Source data are provided as a Source Data file. **f** Aggregate density of expression data in the vicinity of known mRNA 3’ cleavage sites, across various library types. Top three plots, *Drosophila* data; bottom plot; mouse data. Single cell SMART-seq2 data is globally depleted in the vicinity of cleavage sites, whereas bulk 3’-seq data peak at polyadenylation sites. Single nucleus (10X-3’ kit) data also peak in the vicinity of mRNA 3’ termini, but clusters are shifted ~200–250 nt upstream of 3’-seq data. Single cell sciRNA-seq3 mouse data peak closer to 3’ ends than does the fly 10X-3’ data.

Here, we report that diverse patterns of 3’ isoform variation, including both tandem 3’ UTRs and alternative last exons (Fig. 1a), can be detected in *Drosophila* single cell expression data. We are able to resolve the specific cell types that underlie multiple previously reported tissue-specific 3’ isoform programs, reveal new cell-specific 3’ isoform programs, and provide evidence for multiple candidate trans-acting regulators that may implement broad programs of 3’ isoform variation. Notably, in several settings, isoform-level information of broadly-expressed genes can distinguish cell types, providing a new layer for marker classification. These data open new directions to study the regulation of mRNA processing, and comprise a transcriptome annotation resource for the *Drosophila* community that complements existing cell type annotations.

## Results

### Two classes of *Drosophila* 3’ isoforms are resolved in single cell RNA-seq data

Recently, the Fly Cell Atlas (FCA) consortium conducted large-scale profiling of 17 dissected *Drosophila* tissues and organs, and subjected these to broad community-based annotation efforts involving >100 experts^49^. The resulting *Tabula Drosophilae* is built upon ~580,000 cells and includes ~250 defined cell types with high resolution settings. As in most single cell studies, the main FCA analysis effort aggregated reads across each locus, and therefore did not differentiate isoforms in assigning cell types from marker genes.

The FCA dataset was generated using nuclei and the 10X Genomics 3’ v3.1 kit (sNuc-seq). We manually inspected these data on genome browsers by aggregating data by each of the cell types assigned by the FCA. Genes that expressed a single terminal isoform in our 3’-seq profiling similarly exhibit stable 3’ ends across diverse individual cell types (Supplementary Fig. 1a). However, by inspecting genes that we previously recognized to undergo tandem 3’ UTR-alternative polyadenylation (TUTR-APA, Fig. 1a) in specific tissues, we could see that FCA data revealed individual cell types that express distinctive tandem 3’ UTR isoforms. For example, *mei-P26* is an archetypal gene that utilizes a short 3’ UTR in testis, an intermediate one in ovary, and a set of highly elongated 3’ UTRs in the head. We previously documented this variety of 3’ UTR isoforms using RNA-seq data and Northern blotting^8^ as well as 3’-seq libraries from these dissected tissues^9^. Now, we could find individual cell types in each of these tissues that recapitulate this 3’ UTR diversity (Fig. 1b). Similarly, tissue profiling showed that *Hrb27C* expresses a large number of TUTR-APA isoforms, which are also revealed in individual FCA cell types (Supplementary Fig. 1b).

Besides TUTR-APA 3’ isoforms, a substantial set of genes express alternative last exon isoforms (ALE-APA, Fig. 1a). These comprise a type of alternative splicing, but in some cases, the isoform choice is determined via the choice of 3’ cleavage sites^40,50^. For these genes, ALE-APA can generate isoforms with distinct C-termini and fully non-overlapping 3’ UTRs. We noticed that ALE isoforms could also be easily distinguished in single cell data. For example, *shaggy* (*sgg*) is subject to complex 3’ processing including three major ALE-APA isoforms, several of which also undergo TUTR-APA (Fig. 1c). While standard experimental techniques using rt-PCR from dissected tissues cannot resolve the cell-of-origin of tissue-specific 3’ isoforms, we were able to confirm distinct utilization of expected TUTR-APA and ALE-APA isoforms in head, ovary and testis for *mei-P26* and *sgg* (Fig. 1d, e, respectively). The power of single cell approaches to reveal cell-specific APA patterns encouraged us to conduct detailed analyses of both 3’ isoform classes in the FCA data.

### Characteristics of 3’-directed clusters in 10X Genomics data that permit reliable assessment of 3’ isoforms

It is expected that RNA-seq protocols that are not designed to precisely recover polyadenylated junctions will tend to undersample transcript termini. For example, aggregate analysis of FCA SMART-seq2 data shows severe depletion within the last few hundred nucleotides (nts) of mRNA, based on cleavage sites defined from our 3’-seq atlases^9^ (Fig. 1f). Even though the 10X Genomics kit is biased to capture 3’ ends, individual FCA 3’ UTR clusters were some distance upstream of experimentally determined cleavage sites (Fig. 1b, c).

Nevertheless, we reasoned that such upstream shifts may still be acceptable to quantify 3’ isoforms, as long as they exhibit characteristic shifts. We plotted the distribution of 10X data in the vicinity of sites from our 3’ atlas, and found that aggregate FCA data were reliably shifted ~100-300 nt upstream of experimentally determined cleavage sites (Fig. 1f). A recent analysis of sciRNA-seq3 data reported that aggregate clusters peaked ~50 nt upstream of characterized mRNA cleavage sites^36^, which is closer than with 10X FCA data. We recapitulated this result using our analysis pipeline (Fig. 1f). The reason for the greater shift between 10X Genomics and sciRNA-seq3 data is not clear, since the fragment sizes are similar (10X v3.1: 91 bp; sciRNA-seq3: 100 bp) and both strategies involve paired-end sequencing but use only R2 for alignment. In any case, as the 10X Genomics platform is utilized much more frequently than sciRNA-seq3, we reason that the advantage of sciRNA-seq3 data for more precise mapping of 3’ ends is countered by the sheer abundance of 10X Genomics data. In particular, since there is still a long tail of read distribution in sciRNA-seq3 data, the recent APA study elected to parse sciRNA-seq3 only in the vicinity of known 3’ termini, and grouped reads from −200 to +20 of cleavage sites to individual 3’ termini^36^. Since 10X-3’ clusters also exhibit a predictable shift with respect to 3’ ends, a similar approach to analyze the FCA data seemed justified.

In principle, the enhanced cellular resolution of single cell data might permit the study of unannotated, cell-restricted, isoforms that were not apparent in bulk cell/tissue analyses. Inspection of mapped FCA data showed numerous clusters within 3’ UTRs or other gene body regions that were not associated with annotated 3’ ends (e.g. Supplementary Fig. 1b). While some of these might correspond to additional APA or intronic polyadenylation (IPA) sites (which might generate unannotated ALE isoforms), in practice we found these challenging to interpret. First, an enhanced frequency of intronic clusters from non-specific dT priming on primary transcripts is expected for nuclear sequencing^51^. Second, as introns sometimes lie close to transcript termini, some 10X clusters that genuinely reflect 3’ ends appear within coding regions that can be separated from the 3’ UTR by an intron (Supplementary Fig. 1c). Third, certain loci exhibited substantial populations of antisense reads that were not obviously related to known transcription (Supplementary Fig. 1d). Although these might reflect unrecognized transcripts, antisense reads are known artifacts of single cell and 10X data^52,53^. Since a substantial number of *Drosophila* gene models overlap, particularly within their 3’ UTRs^54^, this raised further concerns for interpreting sNuc-seq clusters that were not in the vicinity of known 3’ ends.

For these reasons, we did not further attempt de novo discovery of 3’ termini in the FCA 10X data. Instead, we focused analyses on 10X FCA clusters in the vicinity (up to 300 nts upstream) of 3’ ends in our extensive reference atlas of *Drosophila* mRNA cleavage sites^9^.

### Global analysis of 3’ isoforms across annotated *Drosophila* cell types

With an appreciation of how best to leverage sNuc-seq data to elucidate cell-specific 3’ APA and ALE isoforms, we proceeded to a systematic analysis of the FCA data. We recently used LABRAT to quantify alternative 3’ isoform usage^40,55^, and we since updated this package to handle single cell transcriptomic data (LABRATsc)^56^. We used LABRAT to analyze the aggregated data in each cell cluster, and reserved LABRATsc for single-cell level quantifications in UMAP plots shown in subsequent Figs. We began with our previous comprehensive annotations of experimentally defined *Drosophila* polyadenylation sites^9^, and analyzed sNuc-seq reads within 300 nt of annotated 3’ ends. For each gene in each cell type, we calculated its psi (*ψ*) value, which reflects the relative usage of 3’ isoforms. A *ψ* value of 0 indicates exclusive usage of the most upstream pA site, whereas *ψ* values of 1 indicate exclusive usage of the most downstream pA site. This quantification strategy is easily generalized to handle genes with more than 2 pA sites. For example, for a gene with 3 pA sites, exclusive usage of the middle site would yield a *ψ* value of 0.5. In all cases, a single *ψ* value is assigned to a gene without the need to do multiple pairwise comparisons between pA sites; smaller *ψ* values indicate more usage of upstream pA sites and larger *ψ* values indicate more usage of downstream pA sites.

**Fig. 2:**
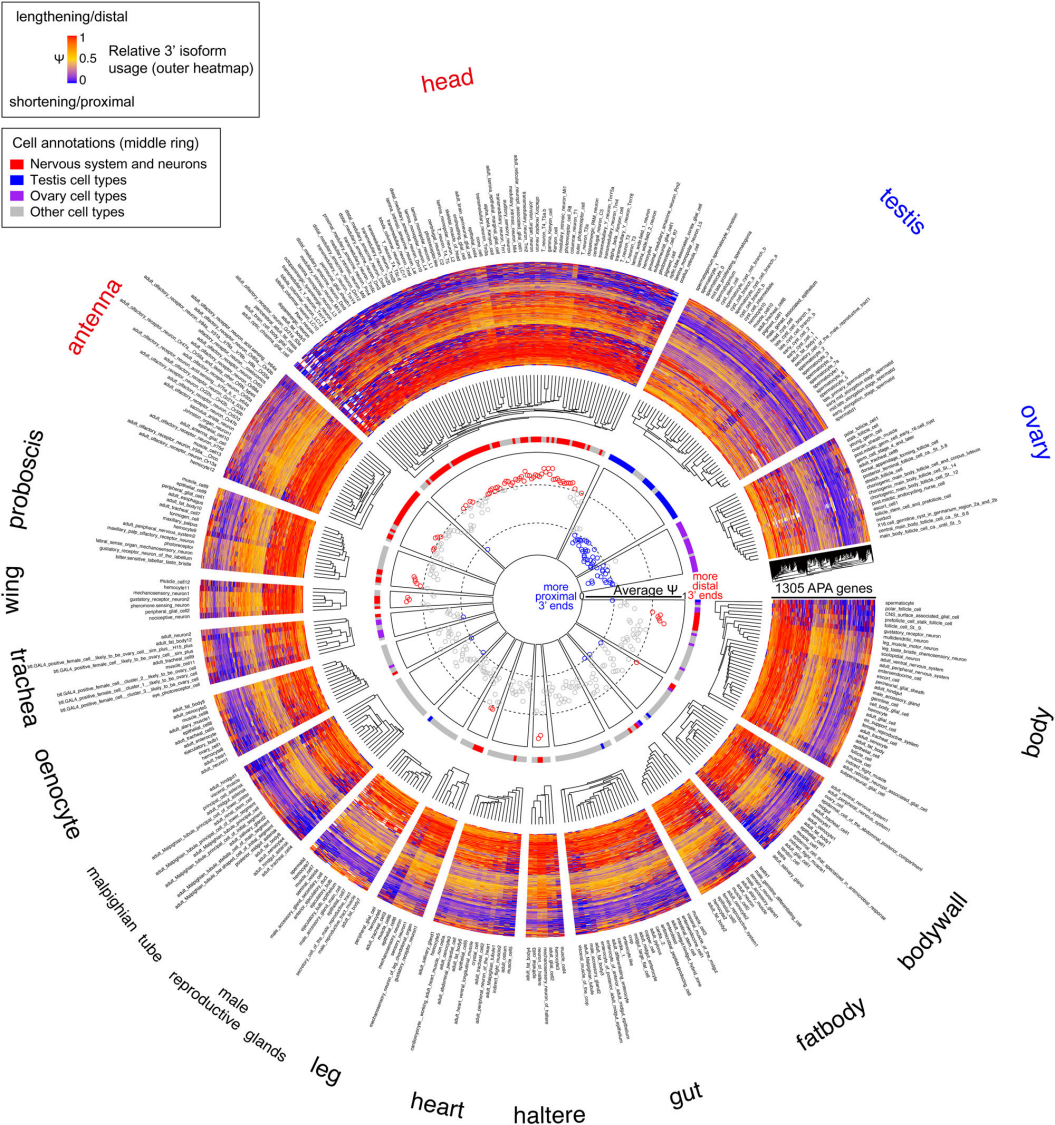
Summary of global 3’ UTR utilization across ~250 *Drosophila* cell types. The circular plot summarizes APA trends across 1305 genes that exhibit differential 3’ UTR utilization from neurons, ovary, and testis cells. We used the LABRAT package to calculate *ψ* across these genes in each of the 242 adult Drosophila cell types annotated by the FCA, grouped according to their tissue origin. Because some cell types are found in multiple tissues, the number of sectors is greater than 242 (e.g., muscle cells, epithelial cells, hemocytes and fat body are present broadly across tissues). We describe the plot features from outside in. The tissue and cell types are shown at the perimeter. Inside this, the behavior of individual genes is shown in heatmap sectors, using the *ψ* color key shown in the center. The cell types in each tissue are organized by hierarchical clustering. The next ring inside highlights selected general cell types in these colors: neurons (red), testis (blue) and ovary (purple). Finally, the inner sectors plot the average *ψ* value (re-scaled 0 to 1, two grey dashes demarcate 0.3 and 0.7, respectively) for each cell type across the selected APA genes. From these analyses, several general features regarding cell type-specific APA are evident. Of note, neurons globally express longest/most distal 3’ UTRs of all cell types (red). Reciprocally, the testis contains cell types that utilize the shortest/most proximal 3’ UTRs of all cell types (blue).

Figure 2 provides a birds-eye view of the analysis. For this high-level perspective, we used 1305 genes that show APA isoform usage differences in one or more settings with distinctive APA landscapes (neurons, ovary, and testis cells), with alteration in average *ψ* value change of >20% between cell types. This circular plot displays hierarchical clustering of constituent cell types of each of the 16 tissue datasets, by their *ψ* values across genes with alternative 3’ ends. We color-coded individual genes based on *ψ* value on a red (1) to blue (0) gradient, and also plotted the average *ψ* value for each cell type in the central portion of the schematic.

Even with these levels of data compression, several broad trends are easily visible, and clearly relate to our previous 3’ atlases based on tissue profiling^9^. For example, certain tissues stand out as having large sectors of cell types bearing “red” genes, reflecting their bias to express distal 3’ isoforms. The most striking tissues are the head and antenna (Fig. 2), which have the largest numbers of annotated neuron types across *Drosophila* tissues^49^. Thus, neurons are the predominant contributor to the expression of highly extended 3’ UTRs across the fly, a situation previously inferred from sequencing of bulk tissues^8,9^.

Conversely, the testis is a tissue with predominant cell type sectors of “blue” genes, meaning that they are biased to express proximal 3’ isoforms. This is easily visualized in the inner circle plot where the overall *ψ* status shows that testis cell types comprise the shortest of all adult *Drosophila* cell types. This is consistent with our previous observation that the testis broadly expresses short 3’ UTRs^8,9^. When inspecting the inner ring summarizing overall *ψ* values for each cell annotation, we see that other “blue” cell types comprise testis/male germline cells that happen to be present in other tissue dissections, but that the ovary also contains many cells with shortening profiles (Fig. 2). We previously noted that the ovary invokes a shortened 3’ UTR program for distinct genes than testis^9^. Thus, male and female gonadal cell types collectively express the shortest 3’ UTRs of all adult *Drosophila* cell types.

Overall, the FCA dataset provides a rich perspective for the cell type-specific expression patterns of 3’ isoforms. The underlying data for all cell types and genes analyzed in Fig. 2 are provided in Supplementary Data 1. Note that this overview does not exclude any FCA annotations, but some of these are based on very few cells (Supplementary Data 2). Such sparse clusters have less power to discriminate isoform expression especially for lower-expressed genes, but all the data are provided for the benefit of community investigations. We proceeded to examine some of these in greater detail, to highlight how regulated 3’-isoforms can be analyzed in the FCA data. As we document, this approach provides additional layers of information to conventional cell-type expression data.

### Parallel implementation of TUTR-APA and ALE-APA programs in diverse neuron types

**Fig. 3:**
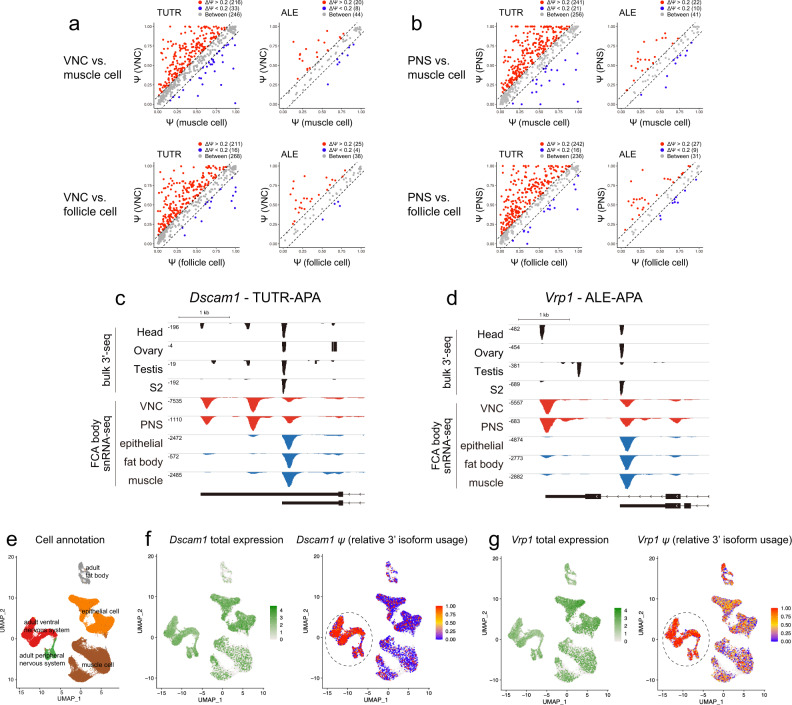
Diverse neuron types broadly implement distal ALE isoforms and extended 3’ UTRs. **a**, **b** Using the body sNuc-seq dataset, we used classifications of ventral nerve cord (VNC) (part of the central nervous system, CNS) and peripheral nervous system (PNS) and compared these to non-neural cell types (muscle and follicle cells shown here, others are shown in Supplementary Fig. 2). In all these comparisons, it is apparent that CNS (**a**) and PNS (**b**) neurons have unidirectional preference to express longer tandem 3’ UTRs as well as distal ALE isoforms. **c**, **d** Genome browser tracks of individual genes highlights that the preferential expression of extended 3’ UTRs (*Dscam1*, **c**) or distal ALE (*Vrp1*, **d**) isoforms in head 3’-seq data is mirrored by their expression in CNS and PNS neurons. **e**–**g** Expression of 3’ isoforms can provide cell type information, where intrinsic gene expression patterns do not. **e** UMAP of dominant cell types in the *Drosophila* body. **f**, **g** APA analysis in individual cells. *Dscam1* and *Vrp1* are broadly expressed genes that do not intrinsically contribute to cell classification. However, expression of the *Dscam1* extended 3’ UTR (**f**) and the *Vrp1* distal ALE isoform (**g**) are indicative of neurons, even in individual cells.

Our prior studies of neural-specific APA were largely inferences, based on the behavior of total RNAs obtained from whole heads or dissected larval CNS^8,9^. As noted, the global APA analysis across all individual cell types (Fig. 2), along with inspection of individual neuron-type RNA-seq tracks (Fig. 1), generally supports the notion that it is indeed neurons that express the distinctive distal ALE isoforms and 3’ UTR extensions, in both the CNS and PNS. We note that PNS neurons have not previously been specifically analyzed for 3’ isoforms in *Drosophila*, since unlike the CNS, PNS neurons are dispersed amongst their constituent tissues and are thus inconvenient for bulk RNA-seq analyses. We examined this further by plotting the behavior of APA genes in various cell types isolated from the body (Fig. 3a, b). This starting material contains both CNS (ventral nerve cord, VNC) and PNS neurons, including other cell types (muscle and follicle cells shown here; selected other cell types are analyzed in Supplementary Fig. 2). These plots show a highly directional shift towards extended tandem 3’ UTR isoforms as well as distal ALE isoforms, in both VNC and PNS neurons, compared to other non-neural cell types. This emphasizes that diverse neuron types express downstream 3’ isoforms compared to many other cell types. Examples of individual genes that express distinctive neural TUTR-APA (*Dscam1*) and ALE-APA (*Vrp1*) isoforms in CNS and PNS are shown in Fig. 3c, d. Lists of genes that undergo TUTR lengthening and/or distal ALE switching in neurons are provided in Supplementary Data 3.

All analyses thus far were based on sNuc-seq data aggregated according to marker genes of cell types defined by the FCA^49^. We further explored whether we could determine specific 3’ isoform usage within individual cells. This places further constraints on gene expression, since there are obviously many dropouts and the numbers of distinct reads that can be mapped to differential isoforms might be relatively few. Nevertheless, a subset of genes consistently express distinct 3’ isoforms at the level of individual cells, particularly in neurons. Examples from the adult body sNuc-seq data are shown in Fig. 3e–g. *Dscam1* and *Vrp1* are relatively broadly expressed, consistent with their behavior in cell-specific sNuc-seq tracks (Fig. 3f, g); thus, these genes would not normally contribute substantially to cell classification. However, most individual VNC and PNS neurons specifically express the 3’ extension of *Dscam1* and the distal ALE isoform of *Vrp1* (Fig. 3f, g). Therefore, there is a subset of broad-expressed genes that do not inform cell type by conventional analysis, but whose isoforms are differentially expressed in discrete cell types and therefore could inform cell identity. Presumably, as sequencing depth increases, the ability to resolve 3’ isoforms in individual cells will similarly increase.

### Highly dynamic expression of TUTR-APA and ALE-APA isoforms in the male germline

**Fig. 4:**
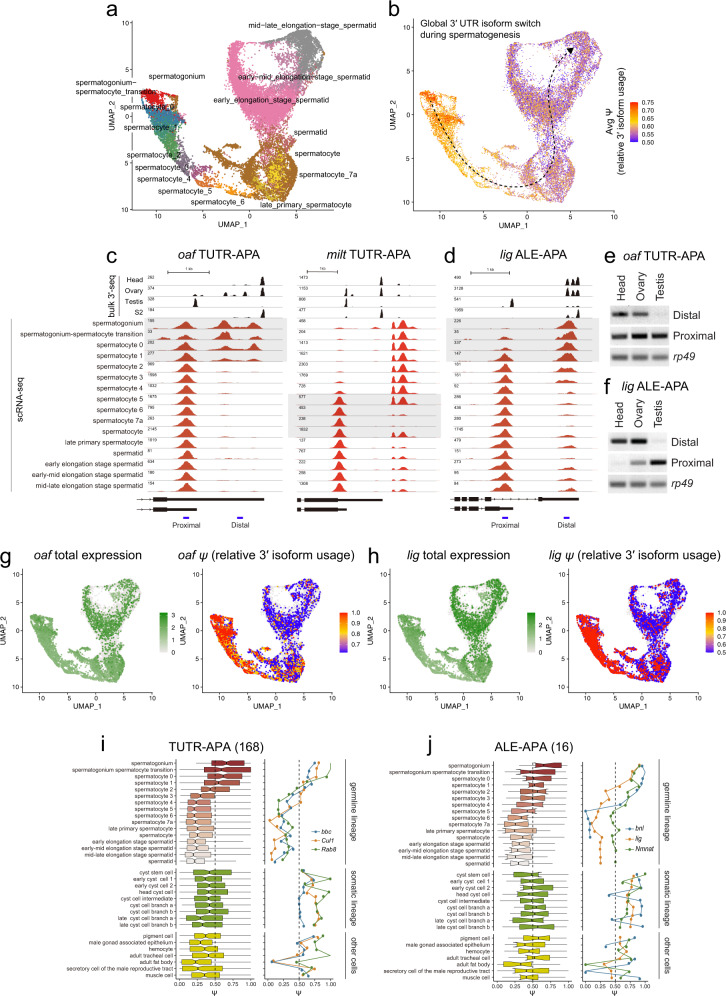
Deconvolution of 3’ isoform programs in the testis germline lineage. **a** UMAP of testis germline cell types in a trajectory from germline stem cells (spermatogonium), meiotic spermatocytes, to post-meiotic spermatids; several intermediary stages are also designated by the FCA. **b** Average 3’ isoform usage of dynamic 3’ isoform switching genes during spermatogenesis in individual cells. **c**, **d** Previously, we observed that bulk testis transcripts exhibit globally shorter 3’ UTRs than most other tissues and cell types (compare with ovary, head and S2 cells), suggesting that a dominant cell type(s) in testis preferentially utilize proximal pA sites. sNuc-seq data pinpoint transitions of 3’ isoforms in the testis germline. **c** Tandem 3’ UTR (TUTR) processing at *oaf*, which utilizes a longer 3’ UTR in spermatogonium and early spermatocyte stages but transitions to a shorter 3’ UTR in spermatocyte stages 1-2, which is maintained into spermatids. **d** Alternative last exon (ALE) processing at *lig*, which utilizes a distal ALE isoform in stem cells but transitions to a proximal ALE isoform in early spermatocytes. **e**, **f** Semi-quantitative RT-PCR validation for testis-specific expression of different 3’ UTR isoforms of *oaf* (**e**) and *lig* (**f**). Data is representative of 2 independent experiments; amplicons are 100–200 bp. Source data are provided as a Source Data file. **g**, **h** APA analysis in individual cells. *oaf* and *lig* maintain similar expression across spermatogenesis. However, expression of the *oaf* extended 3’ UTR (**g**) and the *lig* distal ALE isoform (**h**) are exclusive to early spermatogenesis. **i** Global TUTR shortening in testis occurs during early spermatocyte stages (left, *n* = 168). Examples of TUTR shortening profiles for three representative genes (right) (**j**) Global proximal ALE isoform switching in testis similarly occurs in early spermatocytes (left, *n* = 16). Examples of proximal ALE switching profiles for three representative genes (right). The boxes are shown on the left side of the panels represent the interquartile ranges, the center lines represent medians, and the whiskers denote the ranges of minima and maxima.

Although we were pleased with transcriptome-wide evidence for neurons as the source of elongated 3’ UTRs and distal ALE isoforms, the FCA has a limitation for studying neural 3’ UTR dynamics. As the adult fly head is mostly post-mitotic, except following specific traumatic manipulations^57,58^, this setting is not amenable to study dynamic isoform changes that may occur during the course of cell specification and differentiation. Perhaps a more appropriate setting might be in the germline, where adults continuously generate gametes from germline stem cells (GSCs)^59^. With current FCA data, the ovary is not very promising for this goal, since it lacks sufficient GSCs or annotated oocytes to provide a coherent germline trajectory^49^. However, recent studies characterized testis transcriptomes at single cell resolution^60,61^, and the FCA reveals even greater resolution of cell states within the testis^49^. For example, an initial scRNA-seq study of the adult testis (~5000 cells) divided the male germline into 6 cell types: stem cells/early spermatogonia, late spermatogonia, early/late spermatocytes, and early/late spermatids^61^. Mature spermatids appeared to be depleted, possibly because of their highly elongated shape. In contrast, the FCA datasets contain ~45,000 nuclei, which permitted greater resolution of testis cell states, including during meiosis and subsequent maturation of gametes. For example, the FCA annotated about 40 testis cell types in total and at least 9 spermatocyte-related stages alone^49^ (Fig. 4a).

We analyzed the testis germline lineage in greater detail. Notably, global analysis of 3’ UTR isoforms in individual cells revealed progressive shortening along the lineage from germline stem cell to maturing spermatids (Fig. 4b). We inspected a number of these RNA-seq tracks to ensure that *ψ* values truly reflected the expected shifts in 3’ isoforms, as represented by *oaf* (TUTR-APA) and *lig* (ALE-APA) shown in Fig. 4c, d. For these examples and many others, the isoform switches occurred predominantly in early spermatocyte stages (Supplementary Fig. 3). However, some loci transitioned their 3’ isoforms in later spermatocytes, e.g. *milt* (Fig. 4c). We used conventional rt-PCR analysis of total testis RNA, compared to ovary and head RNA, to confirm dominant trends of TUTR-APA shortening and proximal ALE-APA shifts in testis for *oaf* and *lig* (Fig. 4e, f, respectively). Of note, these loci exhibit strong signatures of 3’ isoform shifts in single cells, even though they are broadly expressed (Fig. 4g, h). This further highlights how isoform information can bolster discrimination of cell states in scRNA-seq data.

When considering all loci undergoing TUTR-APA shortening (*n* = 168) and ALE-APA proximal switching (*n* = 16) in the testis germline in aggregate, the progressive nature of 3’ isoform changes across spermatocyte stages became even more apparent (Fig. 4i, j). We illustrate the behavior of several representative genes in both categories of 3’ isoforms across all testis cell types (germline and somatic) in Fig. 4i, j. Lists of genes that undergo TUTR shifts and/or ALE switching during spermatogenesis are provided in Supplementary Data 4.

Our previous bulk 3’-seq analyses^9^, along with these rt-PCR tests, were suitable for extracting overall trends with large isoform differences between tissues. However, cell-specific resolution of 3’ processing enabled fine dissection of isoform changes. In particular, when analyzing genes with dynamic ALE-APA isoform usage in the testis, we unexpectedly identified three genes that undergo opposite proximal-to-distal 3’ isoform switching in specific spermatocyte stages (Supplementary Fig. 4). Not only is this opposite from the expected distal-to-proximal ALE switching pattern, the timing of these ALE isoform switches was clearly offset, with proximal-to-distal switching occurring during spermatocyte-5 to −7a stages. A particularly complex set of isoform switches is evident at the *SPoCk* locus, which undergoes TUTR shortening and distal-to-proximal ALE switching during the transition from stem cells to early spermatocytes, but then switches back to partial utilization of the distal ALE isoform in late spermatocytes and spermatids (Supplementary Fig. 3b).

Overall, while evidence from genetic manipulations indicated that 3’ UTR shortening occurs in *Drosophila* spermatocytes^62^, this is the first large-scale mapping of 3’ isoforms along the full male germline trajectory. These findings reveal strong parallels with mammalian spermatogenesis, where 3’ UTR shortening in spermatocytes and spermatids was previously inferred from temporal analysis of murine testis development^63–65^. Moreover, these observations point to additional unknown mechanisms for 3’ isoform regulation, and more generally, also support the notion of a high multiplicity of spermatocyte states. These are defined not only by differential gene expression^49^ but also by multiple programs of differential mRNA processing (Fig. 4).

### Novel programs of 3’ isoform switches during differentiation of intestinal stem cells

**Fig. 5:**
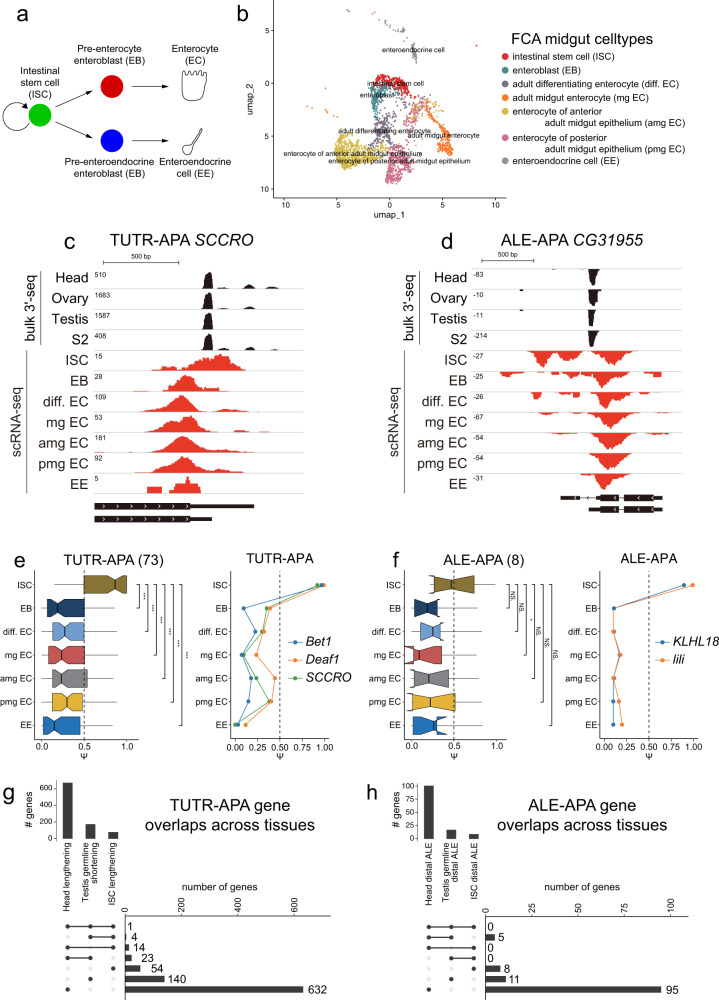
Novel 3’ processing programs in the intestinal stem cell (ISC) lineage. **a** Schematic for differentiation of self-renewing ISCs into enterocytes or enteroendocrine cells. **b** UMAP of the ISC lineage from intestinal FCA data. **c** Example of a gene (*SCCRO*) that undergoes 3’ UTR shortening as ISCs differentiate. **d** Example of a gene (*CG31995*) that undergoes distal-to-proximal ALE switching as ISCs differentiate. Note in both examples (**c**, **d**), neurons do not dominantly express the isoforms found in ISCs, suggesting this reflects a distinct mechanism to adjust 3’ isoform choice in ISCs. **e** Global tandem 3’ UTR lengthening in ISCs (*n* = 73). **f** Global distal ALE isoform usage in ISCs (*n* = 8). The boxes are shown on the left side of the panels represent the interquartile ranges, the center lines represent medians, and the whiskers denote the ranges of minima and maxima. *P < 0.05, **P < 0.01, ***P < 0.001 compared with ISC (*Wilcoxon* test) (**g**, **h**) Overlap analysis amongst neurons, spermatocytes and ISCs confirms that the cell-specific programs of tandem APA (**g**) and ALE-APA (**h**) are substantially distinct across these settings.

Having gained insights into the cell type-specific bases of several tissues associated with distinctive 3’ isoform landscapes, we sought new programs of 3’ isoform regulation. Since stem cell lineages proved auspicious for this purpose, we turned to the intestinal stem cell (ISC) lineage. The *Drosophila* midgut contains multipotent stem cells that maintain the gut during homeostasis and regeneration^66–68^. In particular, ISC division renews the stem cell and/or generates differentiated cell types, namely enteroblasts (EBs) that subsequently yield absorptive enterocytes (ECs), or hormone-producing enteroendocrine (EE) cells (Fig. 5a). All four major cell types were annotated in the FCA data (Fig. 5b); although the EB subtypes were not resolved, several EC subtypes were resolved^49^.

We analyzed 3’ isoform patterns in ISCs and its derivative cell types, and realized that ISCs preferentially express numerous extended 3’ UTRs and distal ALE isoforms compared to their daughter cells. Representative examples with cell-type read coverage are shown in Fig. 5c, d and Supplementary Fig. 5. Based on these observations, we classified 73 genes that shift to shorter TUTR-APA isoforms upon differentiation of ISCs, and 8 genes that shift from distal to proximal ALE-APA isoforms (Supplementary Data 5). We summarize the collective *ψ* value shifts in APA isoforms for both classes of 3’ isoforms, along with illustrative examples of individual genes (Fig. 5e, f). The distinct 3’ isoforms expressed by ISCs are further notable, as it is commonly thought that EBs and ISCs are quite similar in gene expression (Fig. 5b). These dynamic changes in 3’ isoforms are nominally similar to what we observed in the testis germline stem cell lineage, where many genes switch to more proximal 3’ isoforms during stem cell differentiation (Fig. 4). In particular, these trends in *Drosophila* stem cells seem opposite to notions that proliferating cells are correlated with shorter 3’ UTRs^15,69^, perhaps to avoid post-transcriptional regulatory sites. Here in the ISC lineage, as in the male GSC lineage, the multipotent/mitotic stem cell expresses distal 3’ isoforms, which are lost in its differentiated/post-mitotic daughters (e.g., ECs and EEs).

The 3’ isoform programs of ISCs are especially notable as they would have been extremely difficult to parse without single cell transcriptomes. We wondered if it reflected alternative processing of genes that we recognized from other tissue/cell-specific APA settings, or perhaps unannotated loci. Inspection of individual genes showed that some of the isoforms indicative of ISCs were detected in head or testis, but it was not difficult to identify genes for which the dominant 3’ isoform in ISCs was not similarly dominant in these other settings with distinctive APA landscapes. For example, the major extended 3’ UTR isoform of *SCCRO* can be detected in several tissues, but is predominant in ISCs (Fig. 5c), while expression of the distal ALE isoform of *CG31955* is characteristic of ISCs (Fig. 5d). For these loci, heads and neurons do not correspondingly prefer the downstream 3’ isoforms, suggesting that the mechanism of downstream 3’ isoform usage in ISCs is distinct from the recently described strategy for ELAV-mediated downstream 3’ isoform usage in the nervous system^39,40,70^.

To better gauge the tissue-specificity of 3’ isoform shifts, we performed overlap analysis of genes that undergo characteristic TUTR-APA or ALE-APA shifts in neurons, male germline and ISC lineage. Although some genes exhibit reciprocal processing in different settings, the majority of genes are subject to cell-specific 3’ processing events, in both TUTR-APA and ALE-APA classes (Fig. 5g, h). Overall, these observations suggest there are likely multiple mechanisms involved to generate the variety of endogenous cell-specific 3’ isoform landscapes.

### APA programs correlate with specific RBPs

Amongst numerous tissue-specific, perturbation-induced, and/or disease-associated programs and alterations in 3’ isoform programs documented, much remains to be understood about their underlying molecular mechanisms^4,5^. One reasonable inference is that changes in global APA may involve trans-acting regulators. For example, we and others showed that multiple members of the neural-specific ELAV/Hu family of RNA binding proteins (RBPs) are responsible for global induction of 3’ UTR extensions as well as of proximal-to-distal ALE 3’ isoform switching in the nervous system^39,40,70,71^.

Given that neurons have associated functional data on APA mechanisms, we used this as a test case. In a recent mouse scRNA-seq analysis, the global expression of distal 3’ UTRs in neurons was correlated with the elevation of a few RBP families, including ELAV/Hu factors^36^. Accordingly, we assessed FCA data for the presence of distinctive RBPs that were both substantially specific and broadly expressed in neurons (since diverse neuron types alone exhibit strong shifts to 3’ isoforms across adult cell types, Fig. 2), and that were expressed reasonably well (since they presumably need to be abundant enough to rewire transcriptome processing). At the same time, we did not want to fully exclude candidate factors with certain non-neural expression. For example, the ELAV member Rbp9 is capable of inducing neural APA^39^, but in addition to predominant expression in CNS it is also moderately detected in the fat body, where its function is unknown (https://flybase.org/reports/FBgn0010263). In addition, while Elav protein is famously used as neuronal marker, we reported that its transcripts are ubiquitous (albeit to lower levels than in neurons) but restricted by post-transcriptional regulation by miRNAs^72^.

**Fig. 6:**
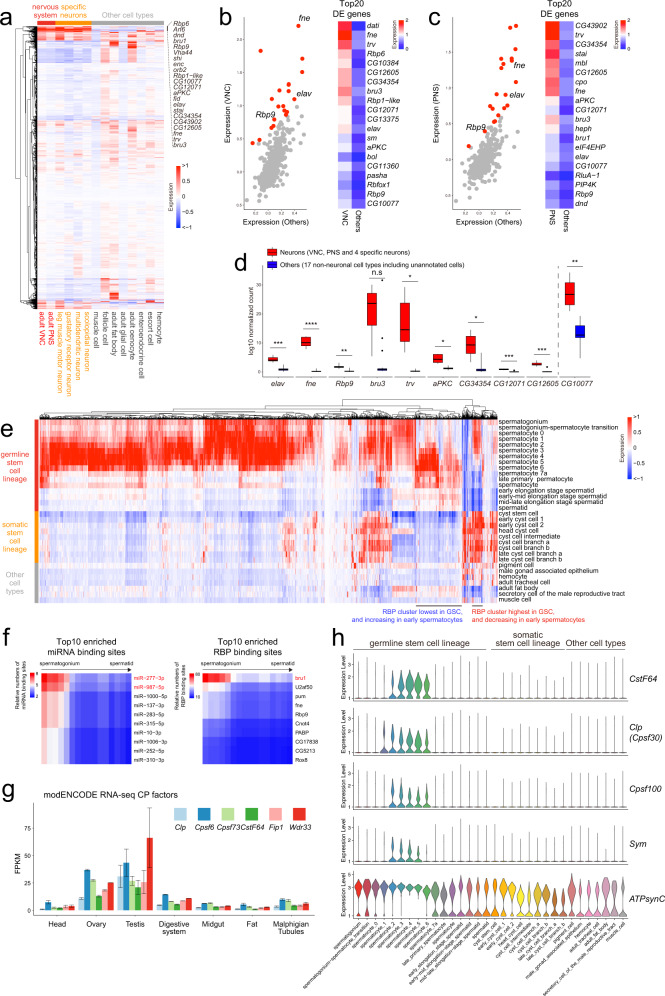
Specific RBPs and global alteration of CPA factors are candidate APA regulators. **a** Hierarchical clustering of RBPs across cell types in the FCA body dataset shows a subset of factors that are commonly elevated across multiple individual neuron types (red and orange cell classes; other cell types designated in gray). These include ELAV/Hu factors Fne and Elav, which direct global neural ALE and APA programs, potentially with their other paralog Rbp9. **b**, **c** Enrichment of RBPs in ventral nerve cord (VNC, part of the central nervous system) and peripheral nervous system (PNS), compared to all other non-neuronal cell types in the FCA body dataset. **d** Expression levels of neural-enriched RBP candidates compared to all other non-neuronal cell types in the FCA body dataset. The boxes represent the interquartile ranges, the center lines represent medians, and the whiskers denote the ranges of minima and maxima. Significant differences between the two groups were assessed by a two-sided Wilcoxon-Wilcox test, with *P* values adjusted from multiple hypothesis testing with a Bonferroni correction. (NS, not significant, *P < 0.05, **P < 0.01, ***P < 0.001, ****P < 0.0001) (**e**) Hierarchical clustering of RBPs across cell types in the FCA testis dataset shows large scale changes in RBP expression in the germline (red cell classes). **f** Top 10 enriched miRNAs (left) and RBPs (right) that target 3’ UTR regions of genes that are lost in shorter isoforms during spermatogenesis. Mostly enriched miRNAs and RBPs are highlighted as red. **g** Multiple cleavage and polyadenylation (CPA) factors are upregulated in gonads compared to all other somatic tissues; data plotted from modENCODE bulk RNA-seq data from dissected tissues. Data are presented as mean values +/− SD pooled from three independent experiments. **h** Data from FCA testis dataset highlights the specific upregulation of multiple CPA factors in early spermatocytes, when global 3’ UTR shortening and proximal ALE switching trends are detected. *ATPsynC* is shown as a ubiquitous control gene that is well-expressed in all germline and somatic cell types of the testis. Data for all CPA factors and additional control genes are shown in Supplementary Fig. 8.

We collected annotated RBPs from EuRBPDB^73^, and supplemented these with additional loci from FlyBase (https://flybase.org) for annotated RNA binding domains (Supplementary Data 6). Hierarchical clustering across the body FCA dataset identified major clusters of neural-enriched RBPs (Fig. 6a and Supplementary Data 7). We plotted the expression of these in both CNS and PNS populations, comparing them to all other non-neuronal body cell types. Although some RBPs were preferentially enriched in one or the other group, a subset was upregulated in both neural populations (Fig. 6b, c). These included ELAV/Hu family RBPs; *fne* and *elav* in particular, with modest enrichment of *rbp9*. To emphasize their expression specificity and differential in neurons vs. other cell types, we compared the levels of these RBPs across multiple neuron types and in non-neural cell types in the body FCA data (Fig. 6d). These other RBPs are candidates to regulate neuronal RNA processing, potentially including selection of 3’ isoforms. Certain factors such as *CG10077* were commonly upregulated in CNS and PNS, but exhibited substantial non-neuronal expression (Fig. 6d); we provisionally consider these as less likely to underlie neural APA given the gain-of-function regulatory properties of ELAV factors^39^. Others seem compelling, such as *bru-3*, a fly CELF gene whose orthologs are also enriched in mammalian neurons^36^.

Similarly, we find that a limited number of RBPs are specifically upregulated in ISCs, and thus correlate with their extended 3’ UTR profile relative to their differentiated progeny (Supplementary Fig. 6). These include several factors that are known to influence mRNA levels and/or processing (e.g., *brat*, *musashi*). These may represent candidates for future studies of the newly-recognized program of cell-specific isoform control in the ISC lineage.

### Testis 3’ shortening correlates with specific miRNAs, RBPs and loss of CPA machinery

In the testis, we documented broad switching from longer to shorter tandem 3’ UTRs, and switching from distal to proximal ALE isoforms (Fig. 4). We identified clusters of RBPs that progressively decrease or increase from spermatogonia and spermatocytes, and are correspondingly low in other somatic cell types (Fig. 6e and Supplementary Data 7). Comparison of stem cells to different spermatocyte stages indicates progressive shifts in RBP expression, particularly amongst those that increase in spermatocytes (Supplementary Fig. 7a and Supplementary Data 8). The changes are particularly overt from spermatocyte 1–3 stages, which is when many 3’ isoforms begin to switch (Fig. 4). Although gene expression is known to generally undergo massive changes at this point in spermatogenesis^60,61,74^, presumably via transcriptional mechanisms^75,76^, the existence of substantial alteration in RBP contents indicates additional regulatory layers that shape the germline transcriptome.

We next sought to identify regulatory consequences of directional APA shifts. We previously analyzed the presence of miRNA and RBP binding sites within 3’ UTR extensions that are predominant within the nervous system^9^. However, we have not previously analyzed a setting of 3’ UTR shortening, such as is characteristic of the male germline. As noted, this is problematic using bulk 3’-seq analysis, since the rarity of male GSCs means that their 3’ UTR status was previously uncharacterized. Using our newly annotated dynamic 3’ UTRs in the male germline (Fig. 4), we searched the 168 3’ UTRs that are specifically longer in GSCs compared to spermatids (Supplementary Fig. 7b) for presence of seed matches to testis-expressed miRNAs^77^ or to RNA binding proteins with defined target sites^78^. This revealed a subset of miRNAs with preferential targeting within GSC 3’ UTR extensions (including miR-277 and miR-987, Fig. 6f and Supplementary Fig. 7c), and a major RBP candidate that preferentially targets these regions (bru1, Fig. 6f and Supplementary Fig. 7d). Notably, examination of individual testis celltypes shows that *bru1* tracks with this APA dynamic, since it is well-expressed in spermatogonium and early spermatocytes, but declines thereafter and is little detected in spermatids (Supplementary Fig. 7e). Therefore, *bru1* appears to selectively target a network of testis transcripts via GSC-specific extended 3’ UTR isoforms. Systematic analysis of miRNA site and RBP sites in testis APA targets are provided in Supplementary Data 9 and 10, respectively.

Among RBPs that increase in early spermatocyte stages, we recognized several components of the core cleavage and polyadenylation (CPA) machinery (Supplementary Fig. 8a). Based on this, we performed a directed analysis of the expression patterns of all CPA factors across testis cell types. While we may expect mRNA processing factors including CPA factors to be ubiquitous, most *Drosophila* cell types exhibit very low levels of CPA factors. However, examination of bulk RNA-seq data across many dissected tissues^79^ shows that CPA factors are particularly high in male and female gonads (Fig. 6g and Supplementary Fig. 8b). We can rationalize the elevated ovary expression as representing maternal deposits. Indeed, most CPA factor transcripts are detected at high levels in embryos prior to zygotic transcription (0–2 h), and their levels usually decline throughout embryogenesis. On the other hand, as paternal contributions to the embryo are more modest than maternal ones, the coordinated elevation of CPA factor transcripts in the testis may be more likely to dynamically affect mRNA processing within the male reproductive system.

To examine this further, we separated testis cell types and plotted their expression of CPA factors. We note a strikingly coordinated increase in most CPA factors in early spermatocytes, precisely when large-scale 3’ isoform changes initiate, and these are maintained through later spermatocyte stages (Fig. 6h and Supplementary Fig. 8c, left). This does not seem to be due to a potential normalization issue involving reorganization of the transcriptome during spermatogenesis, since other presumed housekeeping genes can be robustly detected in all testis germline and somatic cell types, albeit often at higher levels in spermatocytes (Fig. 6h and Supplementary Fig. 8c, right).

Overall, these observations are consistent with the notion that concurrent switching to numerous proximal ALE isoforms and short 3’ UTR isoforms in spermatocytes is facilitated by coordinated elevation of CPA factors, which we hypothesize may enhance cleavage and polyadenylation activity.

## Discussion

### Cellular resolution of APA in *Drosophila*

In this study, we conducted comprehensive analyses of the recently available efforts of the Fly Cell Atlas (FCA) consortium, which profiled 17 dissected tissues to yield ~250 well-defined cell types^49^. This *Tabula Drosophilae* provides a much broader framework to analyze dynamic implementation of 3’ isoforms than we had available using two dozen 3’-seq libraries across *D. melanogaster* stages and tissues^22^, and permits analysis of variable utilization of tandem 3’ UTRs (TUTR-APA) and alternative last exons (ALE-APA). Although a few similar studies of scAPA have recently been reported in mammals^36–38^, our analysis of *Drosophila* also takes advantage of the largest single cell dataset and broadest annotation effort available in this major model organism. In addition, ours is the only study to date to analyze differential usage of ALE 3’ isoforms, which generates distinct coding isoforms with completely different 3’ UTRs amongst isoforms.

This work provides a complementary resource to the main FCA effort to discern cell-specific gene expression, and on this basis, to distinguish cell types^49^. By discovering and leveraging cell-specific patterns of 3’ isoforms, we illustrate many ways in which broadly expressed genes can still contribute to defining cell states, whether they be different types of post-mitotic cells such as neurons and gametes, to stages along the differentiation of various types of stem cells. Undoubtedly, additional patterns of TUTR-APA and ALE-APA remain to be better characterized from these data and approaches. For example, we show that the testis germline stem cell lineage and the intestinal stem cell lineage are replete with coordinated shifts in 3’ isoforms. We note that the digestive system includes ISC, hindgut ISCs, renal and nephric stem cells in Malphighian tubules, and gastric stem cells^80^; which all deserve future investigation. Moreover, although the adult nervous system is largely post-mitotic, application of these approaches during development may permit new insights into neural stem cell lineages and peripheral nervous system lineages^48^. Finally, although the ovary germline lineage is poorly resolved in the FCA, future studies that provide deeper access to ovarian germline stem cells and/or distinguish the transcriptionally silent oocyte from nurse cells, should reveal further 3’ heterogeneity along a relevant developmental trajectory. Importantly, insights can be gained from dispersed cell populations within a tissue, once appropriate knowledge of cell type annotation is in hand.

### Mechanisms of APA and 3’ isoform diversity

Despite the great variety of APA shifts that have been documented in the literature by developmental stages, cell or tissue types, life history traits and perturbations, pathologies and diseases, relatively little is still known about how characteristic APA landscapes are generated. Our study, along with other large-scale profilings, provide a reminder that cell identity is frequently associated with distinct APA isoform landscapes. As these are implicitly driven by genetic programs, at least some of which can be deciphered by correlation analyses with RBPs, we nominate sets of factors whose potential impacts on isoform selection can be rationally interrogated using the abundant targeted genetic tools available in *Drosophila*. Although we focused here on dominant trends, informative 3’ isoform patterns applicable to individual genes can have physiologically relevant consequences. The availability of the complete set of FCA RNA-seq data segregated as individual adult cell type tracks, can fuel future directed studies by community experts. Recent genomic engineering of APA isoform outputs have started to reveal developmental and/or behavioral defects^41,42,81^ and further studies are no doubt upcoming.

Endogenous cell-specific 3’ isoform landscapes are likely to be mimicked by reprogramming of APA isoforms in disease or cancer. Thus, factors that drive endogenous APA changes are prime suspects to be involved in analogous processes during pathological settings. For instance, glioblastoma is associated with lower CFIm25, which in turn correlates with 3’ UTR shortening and tumorigenic properties^82^. A more recent example was the recent demonstration that the testes restricted MAGE-A11 is upregulated in tumors and drives tumorigenesis through ubiquitination of the core pA factor PCF11^83^. This creates a mechanistic connection between the short testis 3’ UTR landscape (i.e., as described in spermatocytes and spermatids) and the short 3’ UTRs characteristic of some proliferative or oncogenic conditions^15,69^. At the same time, caution is warranted if simple edicts apply universally (e.g., that proliferating cells express short 3’ UTRs). For example, in this study, we documented multiple settings where mitotic stem cells exhibit longer 3’ UTR landscapes, but retreat to shorter 3’ UTRs following differentiation. Thus, there are more complex implementations of global APA than currently understood.

Across multiple settings of *Drosophila* APA shifts, we found at least three (neurons, ISCs and in male gametogenesis) where changes across TUTR-APA and ALE-APA isoforms are coordinated in direction. That is to say, that substantial sets of genes prefer internal ALE isoforms and proximal pA sites, or conversely express distal ALE isoforms and extended 3’ UTRs. Several trans-acting factors have been implicated or demonstrated to drive coordinated shifts in TUTR-APA and ALE-APA isoforms^40,84^, and we recently observed global coordination of these 3’ isoform programs across numerous contexts^55^. At the least, this implies that many ALE-APA choices are in fact pA site choices as opposed to splicing choices. However, the underlying mechanistic reason(s) that connect these processes over diverse cell types remain to be better understood.

Of note, many of the gene sets do not overlap amongst settings of global APA that we documented. One possibility is that intrinsic pA site qualities are preferentially organized on gene structures so as to facilitate coordinated shifts. For example, internal pA sites for either APA strategy may be preferentially less effectively cleaved, whereas terminal pA sites in the gene model may harbor optimal features^8,40^. In this scenario, then the efficacy of internal pA site recognition may be generally modulated by, for example, levels of the CPA machinery. The fact that we have found coordinated induction of most of the CPA machinery in early spermatocytes, where large-scale 3’ isoform shifts to internal and/or shorter 3’ UTRs occur, strongly implicates modulation of CPA efficacy to reprogram 3’ isoform landscapes. Experimental knockdown of CPA factors is well known to cause global APA shifts^85–88^, but the effect of upregulating most of all CPA factors remains to be tested directly. More generally, it seems worthwhile to investigate if modulation of specific RBPs might act in concert with specific levels of CPA activity to effect appropriate 3’ isoform programs.

## Methods

### Cell-type specific analysis of the Fly Cell Atlas dataset

Raw sequencing data was retrieved from ArrayExpress (10X Chromium data; accession number: E-MTAB-10519). The fastq files were aligned with CellRanger (version 6.0.1) to an index based on FlyBase genome version 6.28. To split aligned BAM files by annotated clusters, we retrieved cell barcodes associated with each FlyCellAtlas-annotated cell type in each tissue dataset by extracting the annotation column of H5AD datasets from https://flycellatlas.org. We extracted reads that are associated with each cell type and have a mapping score over 30 with Samtools (version 1.12)^89^. Duplicates with an identical UMI and cell barcode were removed with UMI-tools (version 1.0.1)^90^. De-duplicated BAM files were then converted to bigwig files for visualization of coverage with Deeptools (version 3.5.0)^91^. To draw UMAP plots with specific cell types that we focused to compare APA patterns, we used Seurat version 4.0.5^92^ to subtract the cells corresponding to the cell type used in each analysis from the H5AD datasets provided by FlyCellAtlas. RunUMAP function was used to draw UMAP plots with different numbers of PCs according to the number of cell types that we subtracted.

### Quantification of alternative polyadenylation in scRNAseq data

We used two strategies to assign 3’ UTR profiles from scRNA-seq data. For clustering-based quantification, we first aggregated data by each of the cell types assigned by the FCA and considered each of the clusters as a bulk RNA-seq dataset. We then used LABRAT (https://github.com/TaliaferroLab/LABRAT/) to quantify alternative polyadenylation in this data^55^. As LABRAT uses pre-existing transcriptome annotations to quantify the usage of defined polyadenylation sites, we used current *Drosophila melanogaster* gene annotation from FlyBase (version r6.45). Using LABRAT with –librarytype parameter 3pseq, we quantified transcript abundances using only the last 300 nt of annotated transcripts. The expression values are then aggregated to the level of transcript 3’ ends. Expression values for transcripts that have the same or similar (within 25 nt) 3’ ends are summed. LABRAT then reports pA sites usage by comparing the expression values associated with each 3’ end, assigning each gene a *ψ* value. To minimize noise from lowly expressed genes, only genes with at least 100 counts in a sample were analyzed. *ψ* values of 0 indicate exclusive usage of the most proximal pA site, while *ψ* values of 1 indicate exclusive usage of the most distal pA site. LABRAT then identified genes with significantly different *ψ* values across clusters by comparing *ψ* values using a linear model.

To define tandem UTR and ALE gene models, LABRAT observes the isoform structures at the 3’ end of a gene. If all pA sites are contained within the same exon, then the structure is tandem UTR. If all pA sites are contained within different exons, then the structure is ALE. If a gene has more than two pA sites, it is possible for the gene to fit into both classifications. In these cases, LABRAT assigns the gene to have a “mixed” structure. The “mixed” genes were not considered for the analysis specifying “TUTR” and “ALE” models. In this study, 2531 TUTR, 348 ALE and 275 mixed type of genes are analyzed according to the gene structure from the current FlyBase annotation. Of note, TUTR genes harbor total 5445 pA sites (2914 internal and 2531 terminal), and ALE genes harbor total 739 last 3’ exons (391 internal and 348 terminal). We used the R package circlize^93^ to visualize the overall patterns of 3’ UTRs landscape across the entire FCA dataset clusters (Fig. 2).

For single-cell level quantification, we used LABRATsc (https://github.com/TaliaferroLab/LABRAT/tree/singlecell)^56^. LABRATsc works similarly to LABRAT, including defining *ψ* values in a similar way. Reads are first assigned to transcripts using alevin^94^. These transcript-level quantifications are then aggregated to 3’ end-level quantifications. 3’ end-level quantifications are then aggregated across all 3’ ends within a gene to define *ψ* values. Each gene in each cell is therefore assigned a *ψ* value. For each cell, genes with less than one assigned count across all transcripts were excluded from further analysis.

### Analysis of RNA binding protein (RBPs)

For gene expression analysis from 10X Genomics data, we directly downloaded each of 10X Stringent Loom files corresponding to each tissue from FCA website (https://flycellatlas.org/) to access raw counts and clustering information for gene expression analysis. Using default parameters in Seurat version 4.0.5^92^, we log-normalized counts with a scale factor of 10,000 (NormalizeData), and used the normalized values for downstream analyses. For differential expression analysis, the FoldChange function was used to calculate the average difference of each cluster.

We used EuRBPDB^73^, which contains 1633 entries in the *Drosophila* database. This includes 389 non-canonical factors lacking known RNA binding domains, but that are orthologous to proteins identified in RNA interactome capture experiments. Since we noticed that this database lacks some factors associated with RNA binding function, we supplemented this with 78 additional factors from FlyBase that have RBP gene ontology terms (Supplementary Data 6).

### RBP and miRNA binding sites enrichment analysis

We used the FIMO program in MEME Suite (https://meme-suite.org/) to scan RBP sites in the 3’ UTRs with default parameter. Position weight matrices (PWMs) for the RBPs used in this study were reported^78^. TargetScanFly (Release 7.2)^95^ software was used to predict miRNA target sites in the 3’ UTRs. Only strong (8mer and 7mer-m8) miRNA seed matches were considered.

To calculate relative numbers of miRNA/RBP binding sites, each of the numbers derived from above analysis were multiplied with relative usage of alternative polyadenylation sites (*ψ*, LABRAT) to implicate quantitative change in the number of the binding sites in the 3’ UTR according to the relative expression levels of a particular isoform.

### Reverse transcription-Polymerase Chain Reaction (rt-PCR) analysis

We dissected heads, ovaries and testes from <5 day *Drosophila Canton S* adults and prepared total RNA by homogenization in TRIzol. Total RNAs were treated with TurboDNase prior to reverse transcription using SuperScript III (Invitrogen) and analysis on agarose gel. rt-PCR primers designed to amplify 100–200 bp product in length are listed in Supplementary Table 1.

### Reporting summary

Further information on research design is available in the Nature Research Reporting Summary linked to this article.

## Supplementary information

Supplementary Information

Description of Additional Supplementary Files

Supplementary Data 1

Supplementary Data 2

Supplementary Data 3

Supplementary Data 4

Supplementary Data 5

Supplementary Data 6

Supplementary Data 7

Supplementary Data 8

Supplementary Data 9

Supplementary Data 10

Reporting Summary

## Data Availability

The data that support this study are available from the corresponding author upon request. The raw sequencing data and the cell type annotation information generated from the Fly Cell Atlas (FCA) project was used in this study. The 10X Genomics raw data is available at https://www.ebi.ac.uk/arrayexpress/experiments/E-MTAB-10519/. H5AD files for each correspond fly tissues are available at https://flycellatlas.org/. Source data are provided with this paper.

## Source data

Source data

## Code availability

Custom scripts used for analyses in this paper are available at https://github.com/imsjay/scAPA_FCA_paper/tree/main/FlyCellAtlasForAlt3p-main.
