# Supplementary material for: Diverse cell-specific patterns of alternative polyadenylation in Drosophila
Source: Nat Commun. 2022 Sep 13;13:5372. doi: 10.1038/s41467-022-32305-0 (PMC9470587; doi:10.1038/s41467-022-32305-0)
Supplement: Supplementary file 1 — Supplementary Information [file 41467_2022_32305_MOESM1_ESM.pdf]

Supplementary Information for Lee et al,
"Diverse cell-specific patterns of alternative polyadenylation in *Drosophila*"

Full list of Fly Cell Atlas (FCA) Consortium members and affiliations p. 1

Supplementary Figures 1-8 p. 2-9

Fig S1. Illustrative patterns of FCA 10X-3’ data clusters in the vicinity of 3’ UTRs.
Fig S2. Neurons express downstream 3' isoforms relative to other celltypes.
Fig S3. Additional examples of 3’ isoform dynamics in spermatocytes.
Fig S4. Genes that preferentially express distal 3’ isoforms in late spermatocytes.
Fig S5. Additional examples of 3’ isoform shifts in the intestinal stem cell lineage.
Fig S6. RBPs that are differentially expressed in ISCs and their progeny.
Fig S7. RBP and miRNA binding site enrichment analysis.
Fig S8. Most cleavage and polyadenylation (CPA) factors are coordinately induced in early spermatocytes.

Supplementary Table 1 p. 10

FCA participant (total 158)	Last name	Affiliation - 1st	Affiliation - 2nd
Stein Aerts	Aerts	VIB-KU Leuven Center for Brain & Disease Research, KU Leuven, Leuven 3000, Belgium	Laboratory of Computational Biology, Department of Human Genetics, KU Leuven, Leuven 3000, Belgium
Devika Agarwal	Agarwal	MRC Weatherall Institute of Molecular Medicine, University of Oxford, John Radcliffe Hospital, Headington, Oxford OX3 9DS, UK	
Yasir Ahmed-Braimah	Ahmed-Braimah	Department of Biology, Syracuse University, Syracuse NY 13244 USA	
Aaron M. Allen	Allen	Centre for Neural Circuits & Behaviour, University of Oxford, Tinsley Building, Mansfield road, Oxford, OX1 3SR, UK	
Michelle Arbeitman	Arbeitman	Biomedical Sciences Department, Florida State University, Tallahassee, Florida, USA	
Majd M. Ariss	Ariss	Department of Genetics, Harvard Medical School, Boston, MA, USA	
Jordan Augsburger	Augsburger	Department of Cell and Tissue Biology, University of California, San Francisco, CA 94143, USA	
Kumar Ayush	Ayush	Department of Computer Science, Stanford University, Stanford, CA 94305, USA	
Catherine C. Baker	Baker	Department of Developmental Biology, Stanford University School of Medicine, Stanford CA 94305 USA	
Torsten Banisch	Banisch	Skirball Institute and HHMI, New York University Langone Medical Center, New York City, NY 10016, USA	
Cameron W. Berry	Berry	Department of Developmental Biology, Stanford University School of Medicine, Stanford, CA 94305, USA	
Katja Birker	Birker	Development, Aging and Regeneration Program, Sanford Burnham Prebys Medical Discovery Institute, La Jolla, CA 92037 USA	
Rolf Bodmer	Bodmer	Development, Aging and Regeneration Program, Sanford Burnham Prebys Medical Discovery Institute, La Jolla, CA 92037 USA	
Benjamin Bolival	Bolival	Department of Developmental Biology, Stanford University School of Medicine, Stanford CA 94305 USA	
Susanna E. Brantley	Brantley	Department of Developmental Biology, Stanford University School of Medicine, Stanford CA 94305 USA	
Maria Brbić	Brbic	Department of Computer Science, Stanford University, Stanford, CA 94305, USA	
Julie A. Brill	Brill	Cell Biology Program, The Hospital for Sick Children (SickKids), Toronto, ON, M5G 0A4, Canada	Department of Molecular Genetics, University of Toronto, Toronto, ON, M5S 1A8, Canada
Nora C. Brown	Brown	Department of Molecular Biology and Genetics, Cornell University, Ithaca NY 14853, USA	
Katja Brueckner	Brueckner	deceased	
Norene A. Buehner	Buehner	Department of Molecular Biology and Genetics, Cornell University, Ithaca NY 14853, USA	
Xiaoyu Tracy Cai	Cai	Immunology Discovery, Genentech, Inc., 1 DNA Way, South San Francisco, CA 94080, USA	
Rita Cardoso-Figueiredo	Cardoso-Figueiredo	Behavior and Metabolism Laboratory, Champalimaud Research, Champalimaud Centre for the Unknown, Lisbon, Portugal	
Zita Carvalho-Santos	Carvalho-Santos	Behavior and Metabolism Laboratory, Champalimaud Research, Champalimaud Centre for the Unknown, Lisbon, Portugal	
Fernando Casares	Casares	CABD (Andalusian Centre for Developmental Biology), CSIC-UPO-JA. Seville 41013, SPAIN	
Amy Chang	Chang	Department of Molecular and Cell Biology, University of California, Berkeley, Berkeley, CA 94720, USA	
Thomas R. Clandinin	Clandinin	Department of Neurobiology, Stanford University, Stanford, CA 94305, USA	
Sheela Crasta	Crasta	Departments of Bioengineering and Applied Physics, Stanford University, Stanford CA USA, and Chan Zuckerberg Biohub, San Francisco CA USA	
Fabrice David	David	Laboratory of Systems Biology and Genetics, Institute of Bioengineering, School of Life Sciences, Ecole Polytechnique Fédérale de Lausanne (EPFL) and Swiss Institute of Bioinformatics, CH-1015 Lausanne, Switzerland	
Kristofer Davie	Davie	VIB-KU Leuven Center for Brain & Disease Research, KU Leuven, Leuven 3000, Belgium	
Bart Deplancke	Deplancke	*in the main author list, affiliation 8	
Claude Desplan	Desplan	Department of Biology, New York University, New York, New York 10003, USA	
Angela M. Detweiler	Detweiler	Chan Zuckerberg Biohub, San Francisco CA USA	
Darshan B. Dhakan	Dhakan	Behavior and Metabolism Laboratory, Champalimaud Research, Champalimaud Centre for the Unknown, Lisbon, Portugal	
Stephen DiNardo	DiNardo	Perelman School of Medicine, The University of Pennsylvania, and The Penn Institute for Regenerative Medicine Philadelphia, PA 19104 USA	
Erika Donà	Donà	Neurobiology Division, MRC Laboratory of Molecular Biology, Cambridge, CB2 0QH, UK	
Julian A. T. Dow	Dow	Institute of Molecular, Cell & Systems Biology, College of Medical, Veterinary and Life Sciences, University of Glasgow, Glasgow G12 8QQ, UK.	
Stefanie Engert	Engert	Department of Molecular and Cell Biology, University of California, Berkeley, Berkeley, CA 94720, USA	
Swann Floc'hlay	Floc'hlay	VIB-KU Leuven Center for Brain & Disease Research, KU Leuven, Leuven 3000, Belgium	Laboratory of Computational Biology, Department of Human Genetics, KU Leuven, Leuven 3000, Belgium
Margaret T. Fuller	Fuller	Department of Developmental Biology and Department of Genetics, Stanford University School of Medicine, Stanford CA 94305, USA	
Anthony Galenza	Galenza	Department of Molecular and Cellular Physiology, Stanford University School of Medicine, Stanford CA 94305 USA	
Vincent Gardeux	Gardeux	Laboratory of Systems Biology and Genetics, Institute of Bioengineering, School of Life Sciences, Ecole Polytechnique Fédérale de Lausanne (EPFL) and Swiss Institute of Bioinformatics, CH-1015 Lausanne, Switzerland	
Nancy George	George	European Molecular Biology Laboratory, European Bioinformatics Institute, EMBL-EBI, Wellcome Trust Genome Campus, Hinxton CB10 1SD, UK.	
Amanda J González-Segarra	Gonzalez	Department of Molecular and Cell Biology, University of California, Berkeley, Berkeley, CA 94720, USA	
Stephen F Goodwin	Goodwin	Centre for Neural Circuits & Behaviour, University of Oxford, Tinsley Building, Mansfield road, Oxford, OX1 3SR, UK	
Andrew K. Groves	Groves	Department of Molecular and Human Genetics, Baylor College of Medicine, Houston, TX 77030	Department of Neuroscience, Baylor College of Medicine, Houston, TX 77030, USA
Samantha Gumbin	Gumbin	Department of Molecular and Cellular Physiology, Stanford University School of Medicine, Stanford CA 94305 USA	
Yanmeng Guo	Guo	Department of Physiology, Department of Biochemistry and Biophysics, University of California at San Francisco, San Francisco, California, USA	Howard Hughes Medical Institute, San Francisco, California, USA
Devon E. Harris	Harris	Department of Developmental Biology, Stanford University School of Medicine, Stanford CA 94305 USA	
Yael Heifetz	Heifetz	The Hebrew University of Jerusalem, Jerusalem, Israel	
Stephen L. Holtz	Holtz	Department of Neurobiology, Harvard Medical School, Boston, MA 02115, USA	
Felix Horns	Horns	Department of Bioengineering and Biophysics Graduate Program, Stanford University, Stanford, CA 94305 USA	
Bruno Hudry	Hudry	Université Côte d'Azur, CNRS, INSERM, iBV, France	
Ruei-Jiun Hung	Hung	Department of Genetics, Blavatnik Institute, Harvard Medical School, Boston, Massachusetts, USA	
Yuh Nung Jan	Jan	Department of Physiology, Department of Biochemistry and Biophysics, University of California at San Francisco, San Francisco, California, USA	Howard Hughes Medical Institute, San Francisco, California, USA
Jasper Janssens	Janssens	VIB-KU Leuven Center for Brain & Disease Research, KU Leuven, Leuven 3000, Belgium	Laboratory of Computational Biology, Department of Human Genetics, KU Leuven, Leuven 3000, Belgium
Heinrich Jasper	Jasper	Immunology Discovery, Genentech, Inc., 1 DNA Way, South San Francisco, CA 94080, USA	
Jacob S. Jaszczak	Jaszczak	Department of Physiology, Department of Biochemistry and Biophysics, University of California at San Francisco, San Francisco, California, USA	Howard Hughes Medical Institute, San Francisco, California, USA
Gregory S.X.E. Jefferis	Jefferis	Neurobiology Division, MRC Laboratory of Molecular Biology, Cambridge, CB2 0QH, UK	
Robert C. Jones	Jones	Departments of Bioengineering and Applied Physics, Stanford University, Stanford CA USA, and Chan Zuckerberg Biohub, San Francisco CA USA	
Jim Karkanias	Karkanias	Chan Zuckerberg Biohub, San Francisco CA USA	
Timothy L. Karr	Karr	Biodesign Institute, Arizona State University, Tempe AZ 85281, USA	
Nadja Sandra Katheder	Katheder	Immunology Discovery, Genentech, Inc., 1 DNA Way, South San Francisco, CA 94080, USA	
James Kezos	Kezos	Development, Aging and Regeneration Program, Sanford Burnham Prebys Medical Discovery Institute, La Jolla, CA 92037 USA	
Anna A. Kim	Kim	Department of Molecular and Cellular Physiology, Stanford University School of Medicine, Stanford CA 94305 USA	University of California, Santa Barbara, CA 93106, USA
Seung K. Kim	Kim	Department of Developmental Biology, Stanford University School of Medicine, Stanford, CA 94305	Department of Medicine, Stanford University School of Medicine, Stanford, CA 94305, USA
Lutz Kockel	Kockel	Department of Developmental Biology, Stanford University School of Medicine, Stanford, CA 94305, USA	
Sai Saroja Kolluru	Kolluru	Departments of Bioengineering and Applied Physics, Stanford University, Stanford CA USA, and Chan Zuckerberg Biohub, San Francisco CA USA	
Nikolaos Konstantinides	Konstantinides	Institut Jacques Monod, Centre National de la Recherche Scientifique-UMR 7592, Université Paris Diderot, Paris, France	
Thomas B Kornberg	Kornberg	Cardiovascular Research Institute, University of California, San Francisco, CA 94143, USA	
Henry M. Krause	Krause	Donnelly Centre for Cellular and Biomolecular Research, Department of Molecular Genetics, University of Toronto, Toronto, ON, M5S 3E1, Canada	
Andrew Thomas Labott	Labott	Department of Molecular and Cellular Physiology, Stanford University School of Medicine, Stanford CA 94305 USA	
Meghan Laturney	Laturney	Department of Molecular and Cell Biology, University of California, Berkeley, Berkeley, CA 94720, USA	
Ruth Lehmann (confirming)	Lehmann	Skirball Institute, Department of Cell Biology, and HHMI, New York University Langone Medical Center, New York City, NY 10016	current affiliation Whitehead Institute and Department of Biology, MIT, Boston USA
Sarah Leinwand	Leinwand	Department of Molecular and Cell Biology, University of California, Berkeley, Berkeley, CA 94720, USA	
Jure Leskovec	Leskovec	Department of Computer Science, Stanford University, Stanford, CA 94305, USA and Chan Zuckerberg Biohub, San Francisco CA USA	
Hongjie Li	Li	*in the main author list, affiliation 9	
Jiefu Li	Li	Department of Biology, Howard Hughes Medical Institute, Stanford University, Stanford, CA 94305, USA	
Joshua Shing Shun Li	Li	Department of Genetics, Blavatnik Institute, Harvard Medical School, Boston, Massachusetts, USA	
Kai Li	Li	Department of Physiology, Department of Biochemistry and Biophysics, University of California at San Francisco, San Francisco, California, USA	Howard Hughes Medical Institute, San Francisco, California, USA
Ke Li	Li	Department of Physiology, Department of Biochemistry and Biophysics, University of California at San Francisco, San Francisco, California, USA	Howard Hughes Medical Institute, San Francisco, California, USA
Liying Li	Li	Department of Physiology, Department of Biochemistry and Biophysics, University of California at San Francisco, San Francisco, California, USA	Howard Hughes Medical Institute, San Francisco, California, USA
Tun Li	Li	Department of Physiology, Department of Biochemistry and Biophysics, University of California at San Francisco, San Francisco, California, USA	Howard Hughes Medical Institute, San Francisco, California, USA
Maria Litovchenko	Litovchenko	Laboratory of Systems Biology and Genetics, Institute of Bioengineering, School of Life Sciences, Ecole Polytechnique Fédérale de Lausanne (EPFL) and Swiss Institute of Bioinformatics, CH-1015 Lausanne, Switzerland	
Han-Hsuan Liu	Liu	Department of Physiology, Department of Biochemistry and Biophysics, University of California at San Francisco, San Francisco, California, USA	Howard Hughes Medical Institute, San Francisco, California, USA
Yifang Liu	Liu	Department of Genetics, Blavatnik Institute, Harvard Medical School, Boston, Massachusetts, USA	
Tzu-Chiao Lu	Lu	Huffington Center on Aging, Department of Molecular and Human Genetics, Baylor College of Medicine, Houston, TX 77030, USA	
Liqun Luo	Luo	Department of Biology, Howard Hughes Medical Institute, Stanford University, Stanford, CA 94305, USA	
Sharvani Mahadevaraju	Mahadevaraju	Laboratory of Cellular and Developmental Biology, National Institute of Diabetes and Kidney and Digestive Diseases, National Institutes of Health, Bethesda, MD, 20892, USA	
Jonathan Manning	Manning	European Molecular Biology Laboratory, European Bioinformatics Institute, EMBL-EBI, Wellcome Trust Genome Campus, Hinxton CB10 1SD, UK	
Anjeli Mase	Mase	Department of Cell and Tissue Biology, University of California, San Francisco, CA 94143, USA	
Mikaela Matera-Vatnick	Matera-Vatnick	Department of Molecular Biology and Genetics, Cornell University, Ithaca NY 14853, USA	
Neuza Reis Matias	Matias	Department of Developmental Biology, Stanford University School of Medicine, Stanford CA 94305 USA	
Erika L. Matunis	Matunis	Department of Cell Biology, Johns Hopkins University School of Medicine, Baltimore, MD 21205 USA	
Caitlin E. McDonough-Goldstein	McDonough-Goldstein	Department of Biology, Syracuse University, Syracuse, NY, USA	Department of Evolutionary Biology, University of Vienna, Vienna, Austria
Aaron McGeever	McGeever	Chan Zuckerberg Biohub, San Francisco CA USA	
Alex McLachlan	McLachlan	Department of Physiology, Development and Neuroscience, University of Cambridge, Downing Street, Cambridge CB2 3DY, UK	
Colleen N. McLaughlin	McLaughlin	Department of Biology, Howard Hughes Medical Institute, Stanford University, Stanford, CA 94305, USA	
Paola Moreno-Roman	Moreno-Roman	Department of Molecular and Cellular Physiology, Stanford University School of Medicine, Stanford CA 94305 USA	
Norma Neff	Neff	Chan Zuckerberg Biohub, San Francisco CA USA	
Megan Neville	Neville	Centre for Neural Circuits & Behaviour, University of Oxford, Tinsley Building, Mansfield road, Oxford, OX1 3SR, UK	
Sang Ngo	Ngo	Department of Molecular and Cellular Physiology, Stanford University School of Medicine, Stanford CA 94305 USA	
Tanja Nielsen	Nielsen	Development, Aging and Regeneration Program, Sanford Burnham Prebys Medical Discovery Institute, La Jolla, CA 92037 USA	
Todd G. Nystul	Nystul	Department of Anatomy, University of California, San Francisco, CA 94143, USA	
Caitlin E. O'Brien	O'Brien	Department of Physiology, Department of Biochemistry and Biophysics, University of California at San Francisco, San Francisco, California, USA	Howard Hughes Medical Institute, San Francisco, California, USA
Lucy Erin O'Brien	O'Brien	Department of Molecular and Cellular Physiology, Stanford University School of Medicine, Stanford CA 94305 USA	
Brian Oliver	Oliver	Laboratory of Cellular and Developmental Biology, National Institute of Diabetes and Kidney and Digestive Diseases, National Institutes of Health, Bethesda, MD 20892, USA	
David Osumi-Sutherland	Osumi-Sutherland	European Bioinformatics Institute (EMBL/EBI), Wellcome Trust Genome Campus, Cambridge, UK	
Mehmet Neset Özel	Özel	Department of Biology, New York University, New York, NY 10003, USA	
Soumitra Pal	Pal	National Center of Biotechnology Information, National Library of Medicine, NIH, Bethesda, MD 20894, USA	
Irene Papatheodorou	Papatheodorou	European Molecular Biology Laboratory, European Bioinformatics Institute, EMBL-EBI, Wellcome Trust Genome Campus, Hinxton CB10 1SD, UK.	
Norbert Perrimon	Perrimon	Department of Genetics, Blavatnik Institute, Harvard Medical School, Boston, Massachusetts, USA	Howard Hughes Medical Institute, Boston, Massachusetts, USA
Maja Petkovic	Petkovic	Department of Physiology, Department of Biochemistry and Biophysics, University of California at San Francisco, San Francisco, California, USA	Howard Hughes Medical Institute, San Francisco, California, USA
Clare Pilgrim	Pilgrim	Department of Physiology, Development and Neuroscience, University of Cambridge, CB2 3DY, UK	
Angela Oliveira Pisco	Pisco	Chan Zuckerberg Biohub, San Francisco CA USA	
Teresa Przytycka	Przytycka	National Center of Biotechnology Information, National Library of Medicine, NIH, Bethesda, MD 20894, USA	
Stephen R. Quake	Quake	Departments of Bioengineering and Applied Physics, Stanford University, Stanford CA USA, and Chan Zuckerberg Biohub, San Francisco CA USA	
Carolina Reisenman	Reisenman	Department of Molecular and Cell Biology, University of California, Berkeley, Berkeley, CA 94720, USA	
Carlos Ribeiro	Ribeiro	Behavior and Metabolism Laboratory, Champalimaud Research, Champalimaud Centre for the Unknown, Lisbon, Portugal	
Katja Rust	Rust	Institute of Physiology and Pathophysiology, Department of Molecular Cell Physiology, Philipps-University, Marburg, Germany.	Department of Anatomy, University of California, San Francisco, CA 94143, USA
Wouter Saelens	Saelens	Laboratory of Systems Biology and Genetics, Institute of Bioengineering, School of Life Sciences, Ecole Polytechnique Fédérale de Lausanne (EPFL) and Swiss Institute of Bioinformatics, CH-1015 Lausanne, Switzerland	
Erin Nicole Sanders	Sanders	Department of Molecular and Cellular Physiology, Stanford University School of Medicine, Stanford CA 94305 USA	
Gil dos Santos	Santos	The Biological Laboratories, Harvard University, 16 Divinity Avenue, Cambridge, MA 02138, USA	
Frank Schnorrer	Schnorrer	Aix-Marseille University, CNRS, IBDM (UMR 7288), Turing Centre for Living systems, 13009 Marseille, France	
Kristin Scott	Scott	Department of Molecular and Cell Biology, University of California, Berkeley, Berkeley, CA 94720, USA	
Aparna Sherlekar	Sherlekar	Department of Molecular and Cellular Physiology, Stanford University School of Medicine, Stanford CA 94305 USA	
Jiwon Shim	Shim	Department of Life Science, College of Natural Science, Hanyang University, Seoul, Republic of Korea 04763	
Philip Shiu	Shiu	Department of Molecular and Cell Biology, University of California, Berkeley, Berkeley, CA 94720, USA	
David Sims	Sims	MRC Weatherall Institute of Molecular Medicine, University of Oxford, John Radcliffe Hospital, Headington, Oxford OX3 9DS, UK	
Rene V. Sit	Sit	Chan Zuckerberg Biohub, San Francisco CA USA	
Maija Slaidina	Slaidina	Skirball Institute, Faculty of Medicine, New York University, New York, NY 10016	current affiliation - University of Bergen, Faculty of Medicine, Bergen, Norway
Harold E. Smith	Smith	Genomics Core, National Institute of Diabetes and Digestive and Kidney Diseases, US National Institutes of Health, Bethesda, MD, USA	
Katina Spanier	Spanier	VIB-KU Leuven Center for Brain & Disease Research, KU Leuven, Leuven 3000, Belgium	Laboratory of Computational Biology, Department of Human Genetics, KU Leuven, Leuven 3000, Belgium
Gabriella Sterne	Sterne	Department of Molecular and Cell Biology, University of California, Berkeley, Berkeley, CA 94720, USA	
Yu-Han Su	Su	Department of Molecular and Cellular Physiology, Stanford University School of Medicine, Stanford CA 94305 USA	
Daniel Sutton	Sutton	Graduate Program in Genetics and Genomics, Department of Molecular and Human Genetics, Baylor College of Medicine, Houston, TX, 77030 USA	
Marco Tamayo	Tamayo	Development, Aging and Regeneration Program, Sanford Burnham Prebys Medical Discovery Institute, La Jolla, CA 92037 USA	
Michelle Tan	Tan	Chan Zuckerberg Biohub, San Francisco CA USA	
Ibrahim Tastekin	Tastekin	Behavior and Metabolism Laboratory, Champalimaud Research, Champalimaud Centre for the Unknown, Lisbon, Portugal	
Sudhir Gopal Tattikota	Tattikota	Department of Genetics, Blavatnik Institute, Harvard Medical School, Boston, Massachusetts, USA	
Christoph Treiber	Treiber	Centre for Neural Circuits & Behaviour, University of Oxford, Tinsley Building, Mansfield Road, Oxford, OX1 3TA, UK	
David Vacek	Vacek	Department of Biology, Howard Hughes Medical Institute, Stanford University, Stanford, CA 94305, USA	
Georg Vogler	Vogler	Development, Aging and Regeneration Program, Sanford Burnham Prebys Medical Discovery Institute, La Jolla, CA 92037 USA	
Scott Waddell	Waddell	Centre for Neural Circuits & Behaviour, University of Oxford, Tinsley Building, Mansfield Road, Oxford, OX1 3TA, UK	
Maxime De Waegeneer	Waegeneer	VIB-KU Leuven Center for Brain & Disease Research, KU Leuven, Leuven 3000, Belgium	Laboratory of Computational Biology, Department of Human Genetics, KU Leuven, Leuven 3000, Belgium
Wanpeng Wang	Wang	Cardiovascular Research Institute, University of California, San Francisco, CA 94143, USA	
Helen White-Cooper	White-Cooper	School of Biosciences, Cardiff University, Cardiff, CF10 3AX UK	
Rachel I. Wilson	Wilson	Department of Neurobiology, Harvard Medical School, Boston, MA 02115, USA	
Mariana F. Wolfner	Wolfner	Department of Molecular Biology and Genetics, Cornell University, Ithaca NY 14853, USA	
Yiu-Cheung E. Wong	Wong	Department of Developmental Biology, Stanford University School of Medicine, Stanford CA 94305 USA	
Anthony Xie	Xie	Department of Biology, Howard Hughes Medical Institute, Stanford University, Stanford, CA 94305, USA	
Qijing Xie	Xie	Department of Biology, Howard Hughes Medical Institute, Stanford University, Stanford, CA 94305, USA	
Jun Xu	Xu	Department of Genetics, Blavatnik Institute, Harvard Medical School, Boston, Massachusetts, USA	
Shinya Yamamoto	Yamamoto	Department of Molecular and Human Genetics, Baylor College of Medicine, Houston, TX 77030, USA	Jan and Dan Duncan Neurological Research Institute, Texas Children's Hospital, Houston, TX 77030, USA
Jia Yan	Yan	Chan Zuckerberg Biohub, San Francisco CA USA	
Zepeng Yao	Yao	Department of Molecular and Cell Biology, University of California, Berkeley, Berkeley, CA 94720, USA	
Kazuki Yoda	Yoda	Department of Molecular and Cellular Physiology, Stanford University School of Medicine, Stanford CA 94305 USA	
Ruijun Zhu	Zhu	Department of Physiology, Department of Biochemistry and Biophysics, University of California at San Francisco, San Francisco, California, USA	Howard Hughes Medical Institute, San Francisco, California, USA
Robert P Zinzen	Zinzen	Laboratory for Systems Biology of Neural Tissue Differentiation, Berlin Institute for Medical Systems Biology (BIMSB), Max Delbrueck Centre for Molecular Medicine (MDC) in the Helmholtz Association, Robert-Roessle-Strasse 12, 13125 Berlin, Germany	

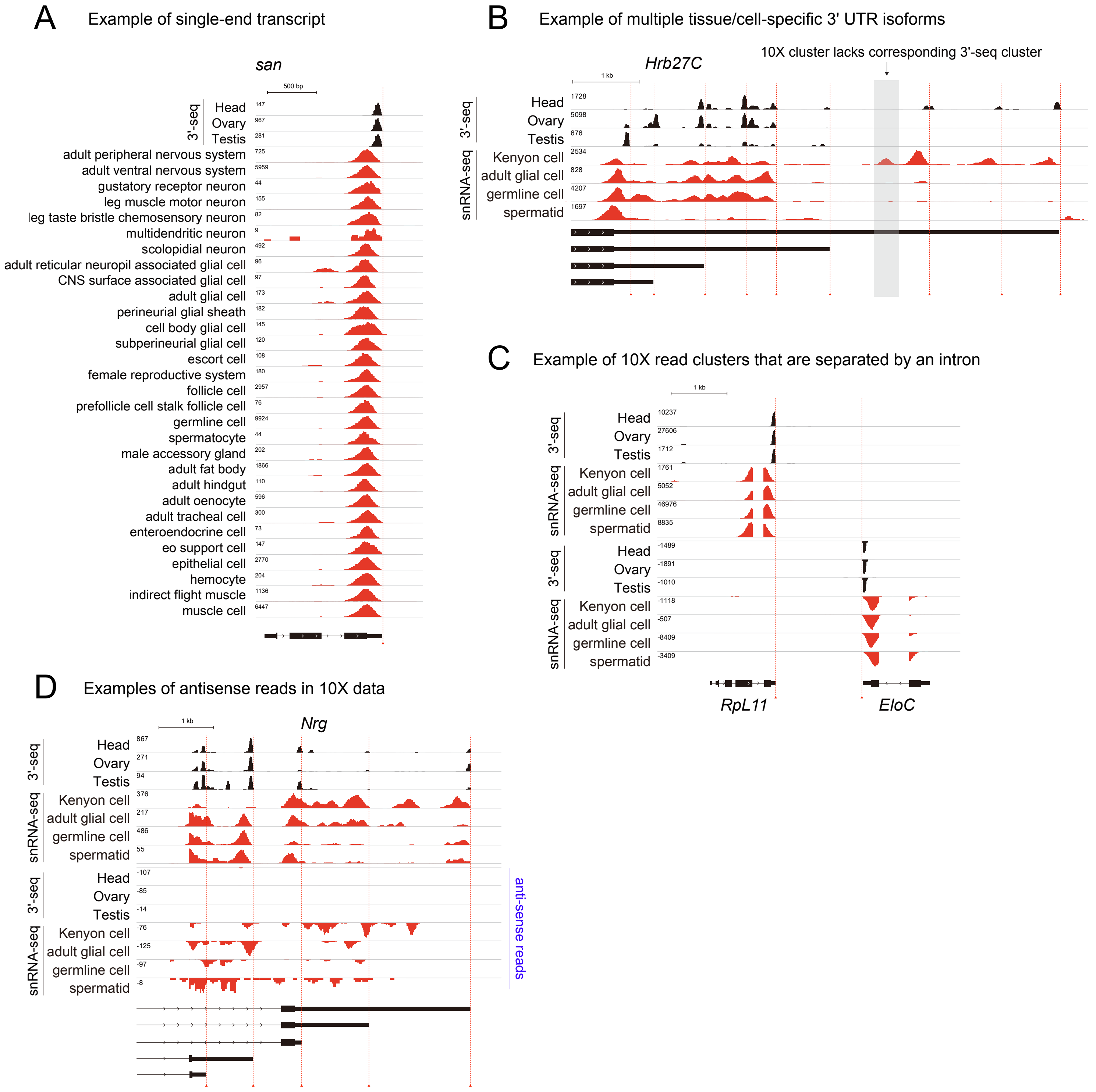

Supplementary Figure 1. Illustrative patterns of FCA 10X-3' data clusters in the vicinity of 3' UTRs.

(A) Many genes stably express a single 3' UTR isoform, as determined by tissue-specific 3'-seq profiling, and this is mirrored in their highly reproducible, non-alternative, 10x read clusters across individual cell types. (B) Example of a gene with multiple tissue-specific tandem 3' UTR isoforms, which are seen in both bulk tissue-level 3'-seq and 10X data. Grey shaded region highlights 10X read clusters that lack corresponding 3'-seq signals. While these might be cell-specific 3' ends, we chose to be conservative and did not consider scRNA-peaks that were not in the vicinity of annotated 3'-seq termini. (C) Because 10x-3' clusters are located upstream of 3' ends, in cases where the terminal exon is very short, the single cell data can be separated from the 3' end by an intron. Thus, read clusters should be interpreted relative to the transcriptome and not the genome. (D) Examples of antisense reads in 10X data. The Nrg locus generates multiple types of ALE-APA and TUTR-APA 3' isoforms. The 3'-seq data indicates largely sense strand data in head/ovary/testis; however, 10X data of constituent cells in these tissues that express these isoforms also shows substantial antisense clusters, which may be an artifact of single cell sequencing.

Supplementary Figure 1
Lee et al

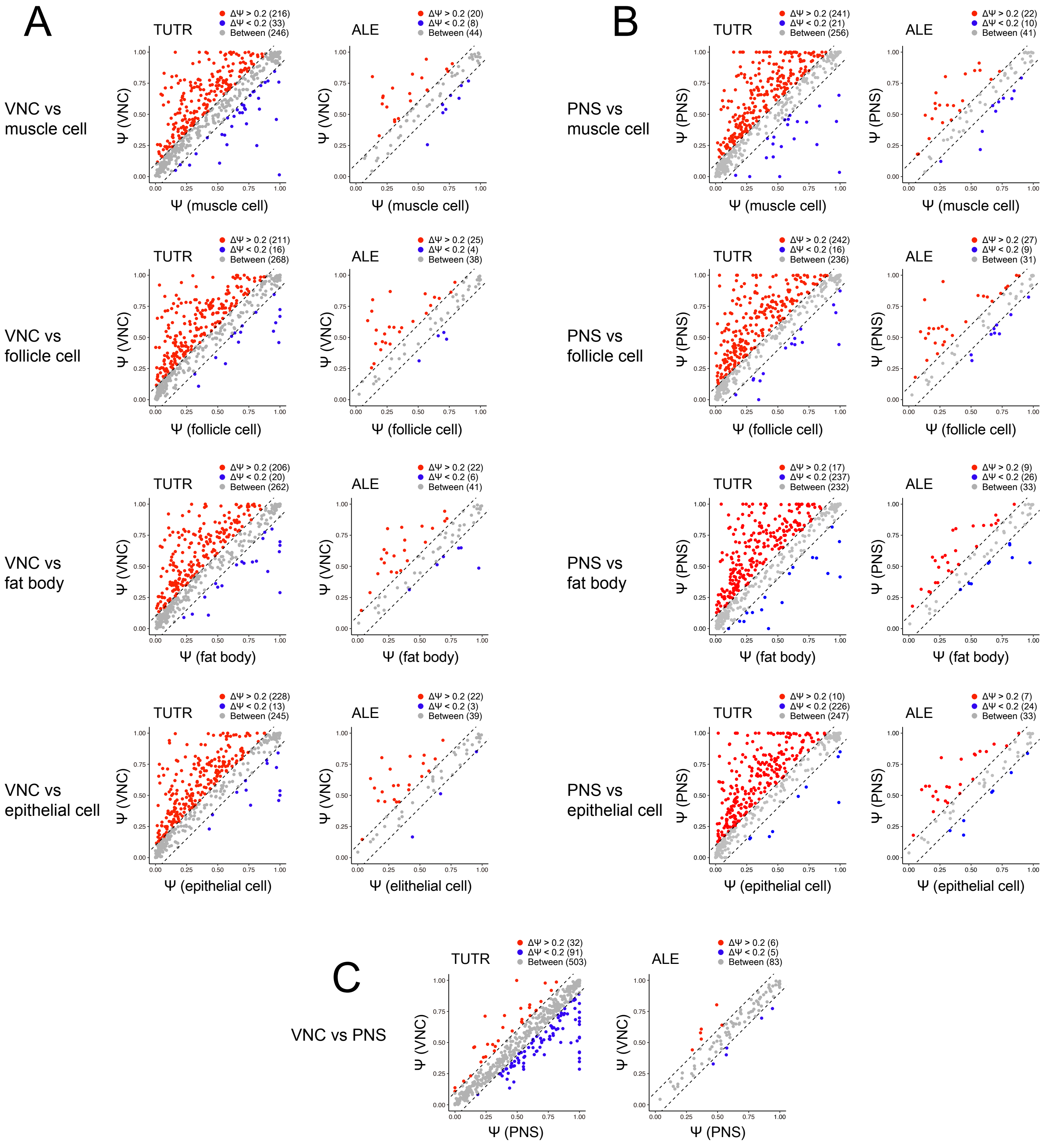

Supplementary Figure 2. Neurons express downstream 3' isoforms relative to other celltypes.

This figure depicts the relative usage of 3' isoforms (PSI) at individual genes in several annotated celltypes from the adult body FCA dataset. (A-C) The left panels in each group represent genes subject to tandem 3' UTR (TUTR) APA, the right panels in each group depict genes subject to alternative last exon (ALE) APA. Genes are colored red if they substantially express more extended/distal 3' isoforms for the Y-axis celltype compared to the X-axis celltype, blue if they have the reciprocal property, and grey if similar between celltypes. The numbers of genes in each category for each comparison are listed in each graph. (A) Comparison of the ventral nerve cord (VNC) to other major body celltypes. (B) Comparison of peripheral nervous system (PNS) to other major body celltypes. (C) Comparison of the VNC and PNS.

Supplementary Figure 2
Lee et al

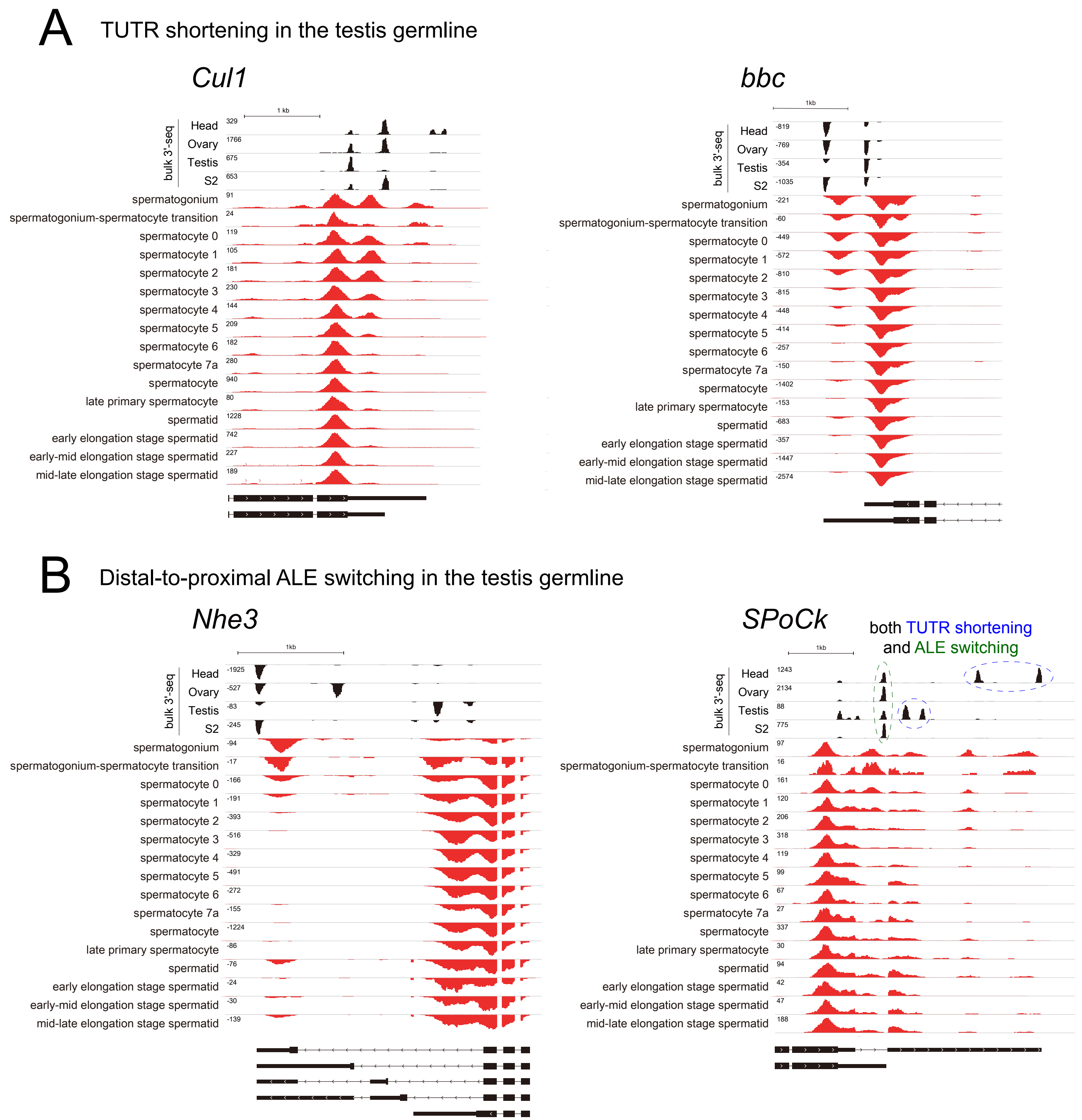

Supplementary Figure 3. Additional examples of 3' isoform dynamics in spermatocytes.

This figure shows IGV browser tracks for bulk 3'-seq data from head/ovary/testis/S2 cells (in black, top tracks) and 10X-3' scRNA-seq data from individual cell types in the testis; gene models are shown at the bottom. (A) Examples of switching from longer to shorter tandem 3' UTRs (TUTR) in early spermatocytes. (B) Examples of switching from distal to proximal alternative last exon (ALE) isoforms in early spermatocytes. Note that SPoCk is subject to both TUTR shortening and ALE switching in the germline lineage, and having lost expression of the distal ALE isoform in favor of the proximal ALE isoform in early spermatocytes, it actually regains partial expression of the distal ALE isoform in late spermatocytes and spermatids.

Supplementary Figure 3
Lee et al

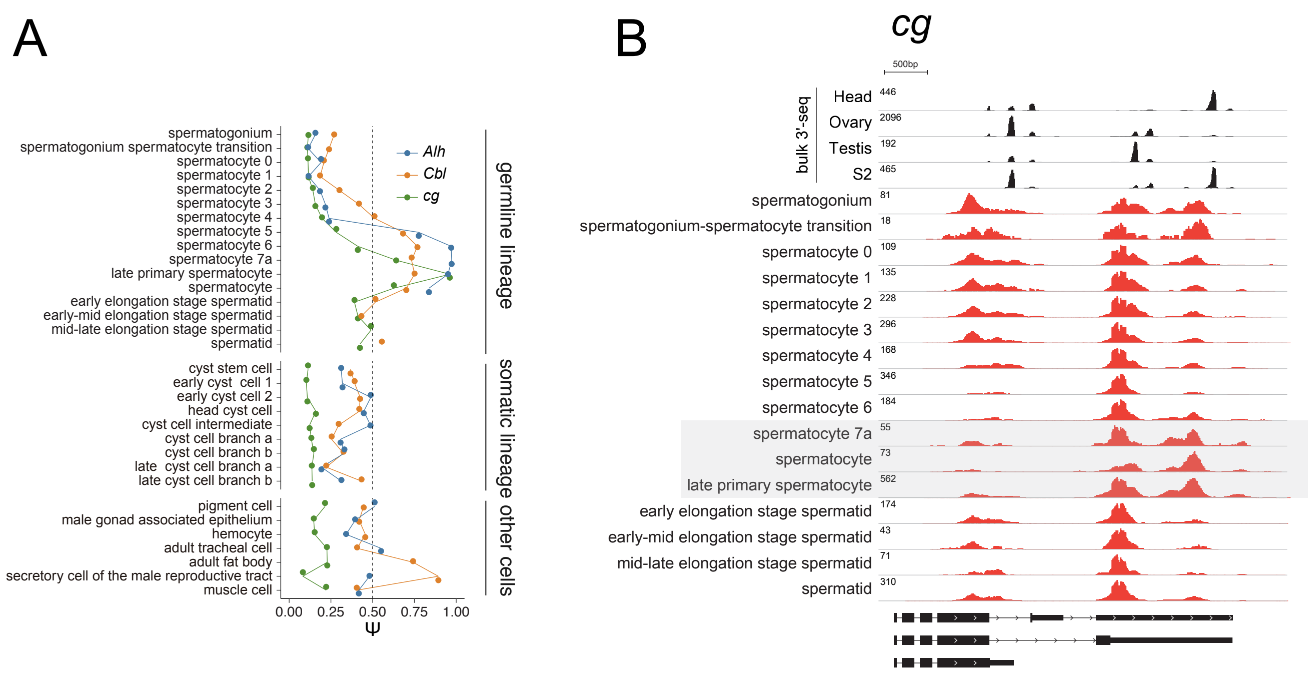

Supplementary Figure 4. Genes that preferentially express distal 3’ isoforms in late spermatocytes.

(A) Although we find global 3’ shortening (both TUTR-APA and ALE-APA) across the testis germline lineage, a subcluster of three genes exhibits opposite proximal-to-distal ALE switching in late spermatocytes. (B) IGV browser tracks illustrating the distal 3’ isoform shifts at *cg* in late spermatocytes. This opposite behavior indicates diverse mechanisms for 3' processing in the testis germline.

Supplementary Figure 4
Lee et al

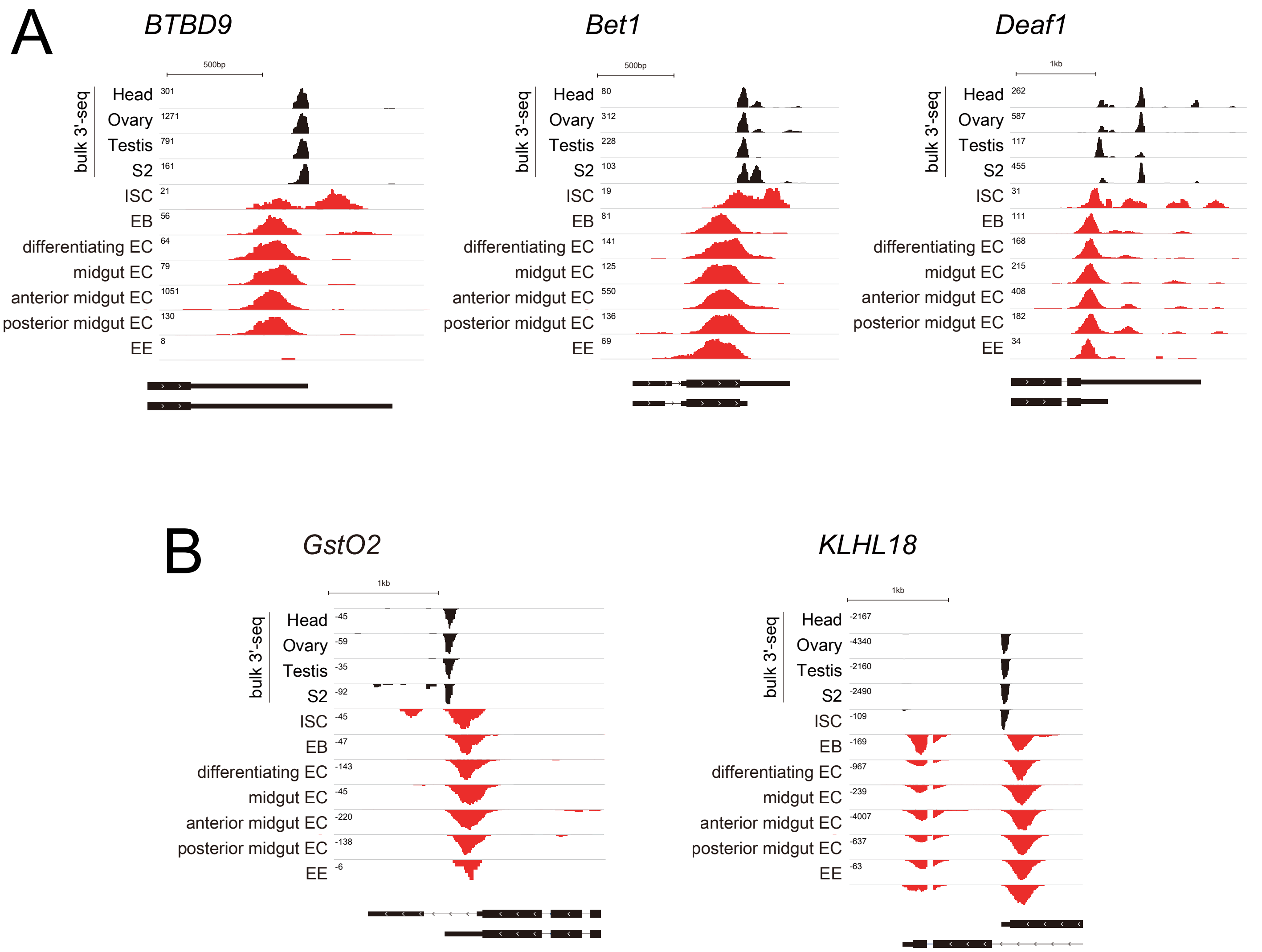

Supplementary Figure 5. Additional examples of 3' isoform shifts in the intestinal stem cell lineage.

This figure shows IGV browser tracks for bulk 3'-seq data from head/ovary/testis/S2 cells (in black, top tracks) and 10X-3' scRNA-seq data from individual cell types in the gut; gene models are shown at the bottom. Intestinal stem cell (ISC), enteroblast (EB), enterocyte (EC), enteroendocrine cell (EE). (A) Examples of switching from longer to shorter tandem 3' UTRs (TUTR) in the differentiated progeny of ISCs. (B) Examples of switching from distal to proximal alternative last exon (ALE) isoforms in the differentiated progeny of ISCs. All of these gut celltypes express some amount of both ALE isoforms, but ISCs express greater relative levels of the distal ALE isoforms of both genes, compared to all other gut celltypes.

Supplementary Figure 5
Lee et al

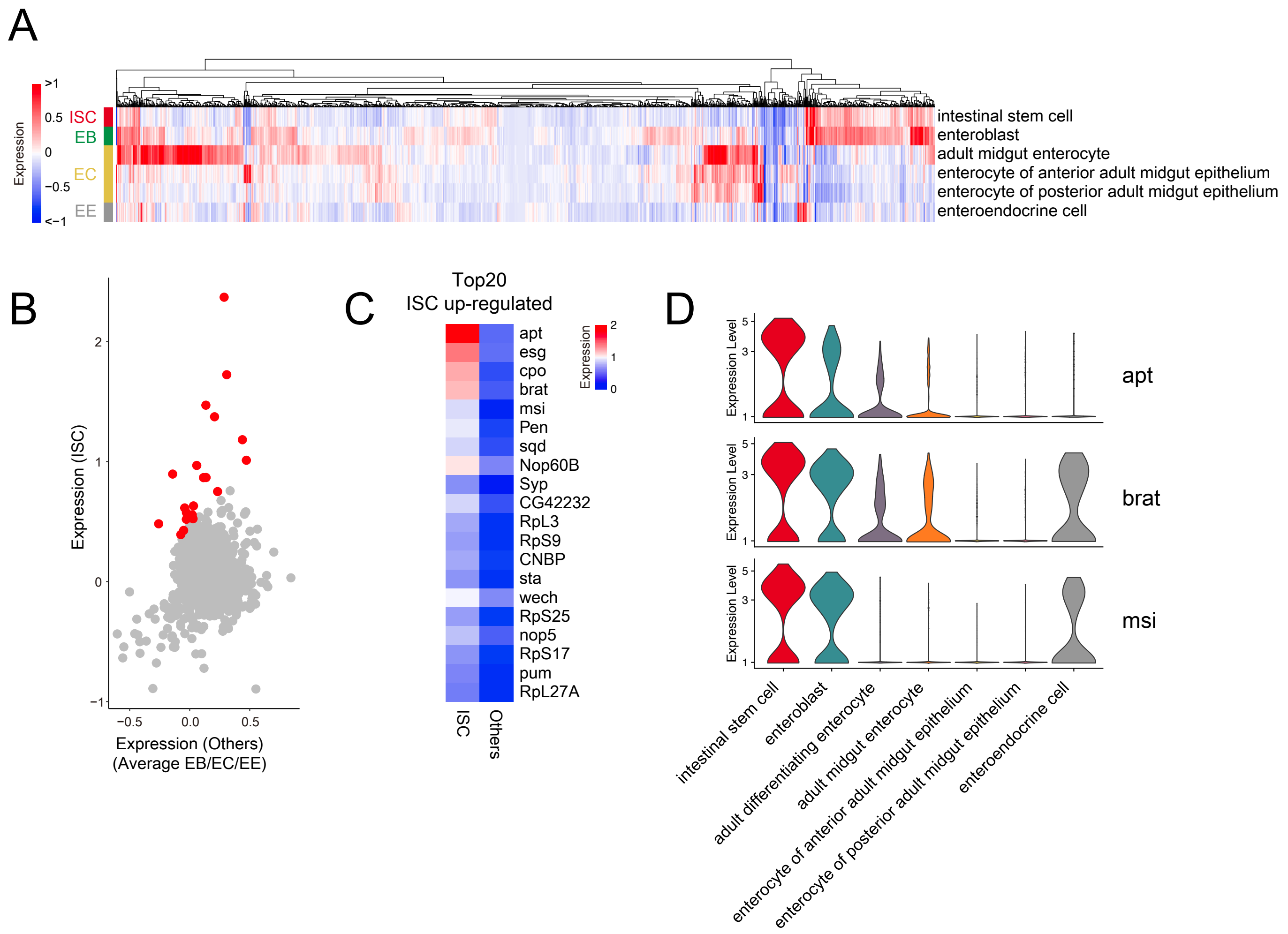

Supplementary Figure 6. RBPs that are differentially expressed in ISCs and their progeny.

(A) Expression of RBPs in the intestinal stem cell (ISC) lineage, which gives rise to enteroblasts (EBs) and enterocytes (ECs), as well as enteroendocrine (EE) cells. (B) RBPs that are upregulated in ISCs compared to other lineage cell types. (C) Heat map of the top 20 RBPs that are upregulated in ISCs. (D) Violin plots showing the expression of RBP candidates that are highest in ISCs and/or their immediate progeny (EBs).

Supplementary Figure 6
Lee et al

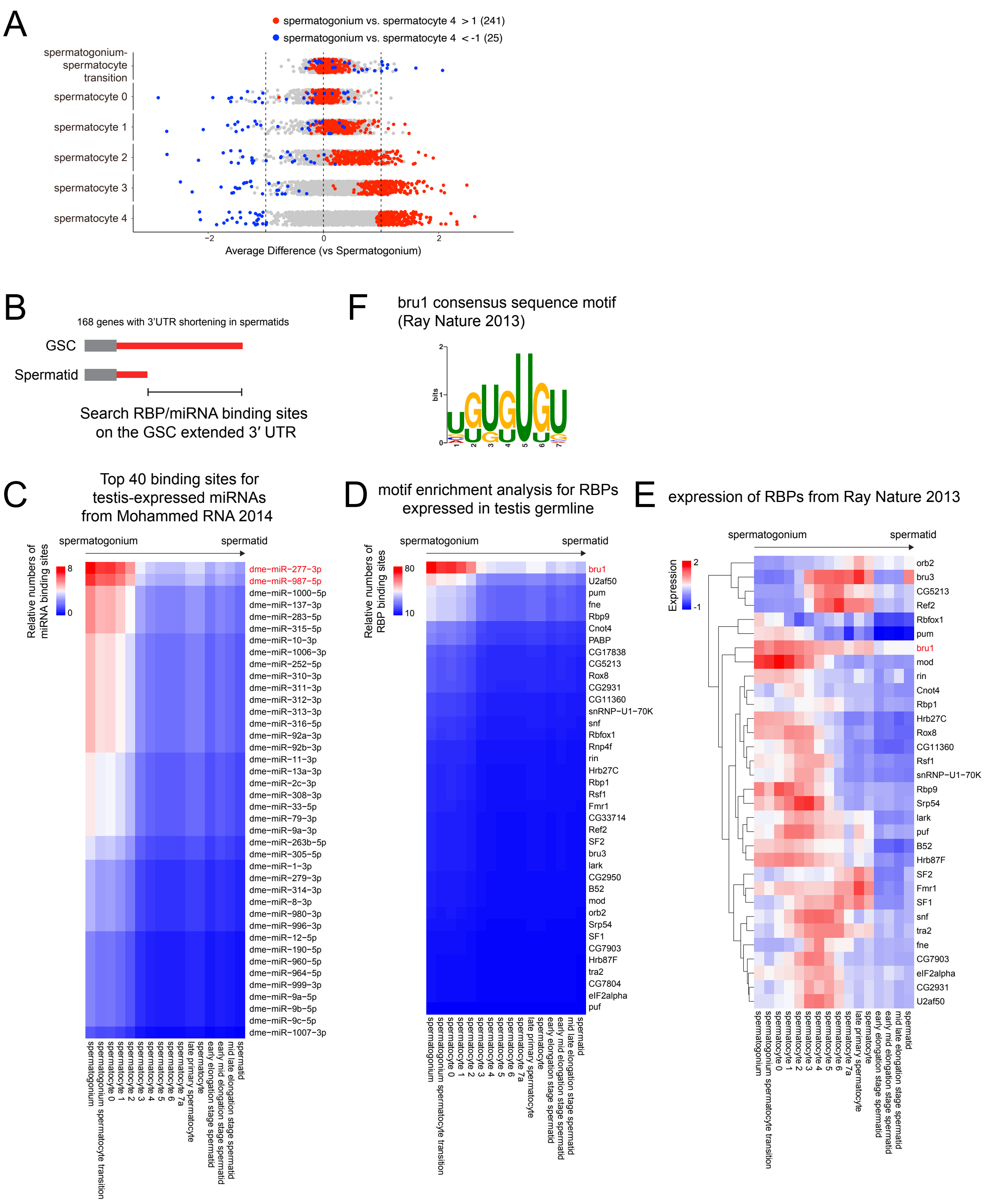

Supplementary Figure 7. RBP and miRNA binding site enrichment analysis.

(A) Comparison of RBP expression in various early spermatocyte stages with the spermatogonium (germline stem cell) stage emphasizes a dominant trend of RBP induction. (B) Strategy to evaluate regulatory impacts of directional 3'UTR dynamics in the testis germline. (C) Conserved miRNA sites for testis-expressed miRNAs enriched within GSC-extended 3' UTRs, included miR-277 and mir-987 as top hits. (D) RBP sites within GSC-extended 3' UTRs shows bru1 as the predominant hit. (E) Expression of RBPs from (D) in testis germline cell types shows that bru1 is correspondingly enriched in GSCs and is lost during differentiation. (F) Reported Bru1 binding site.

Supplementary Figure 7
Lee et al

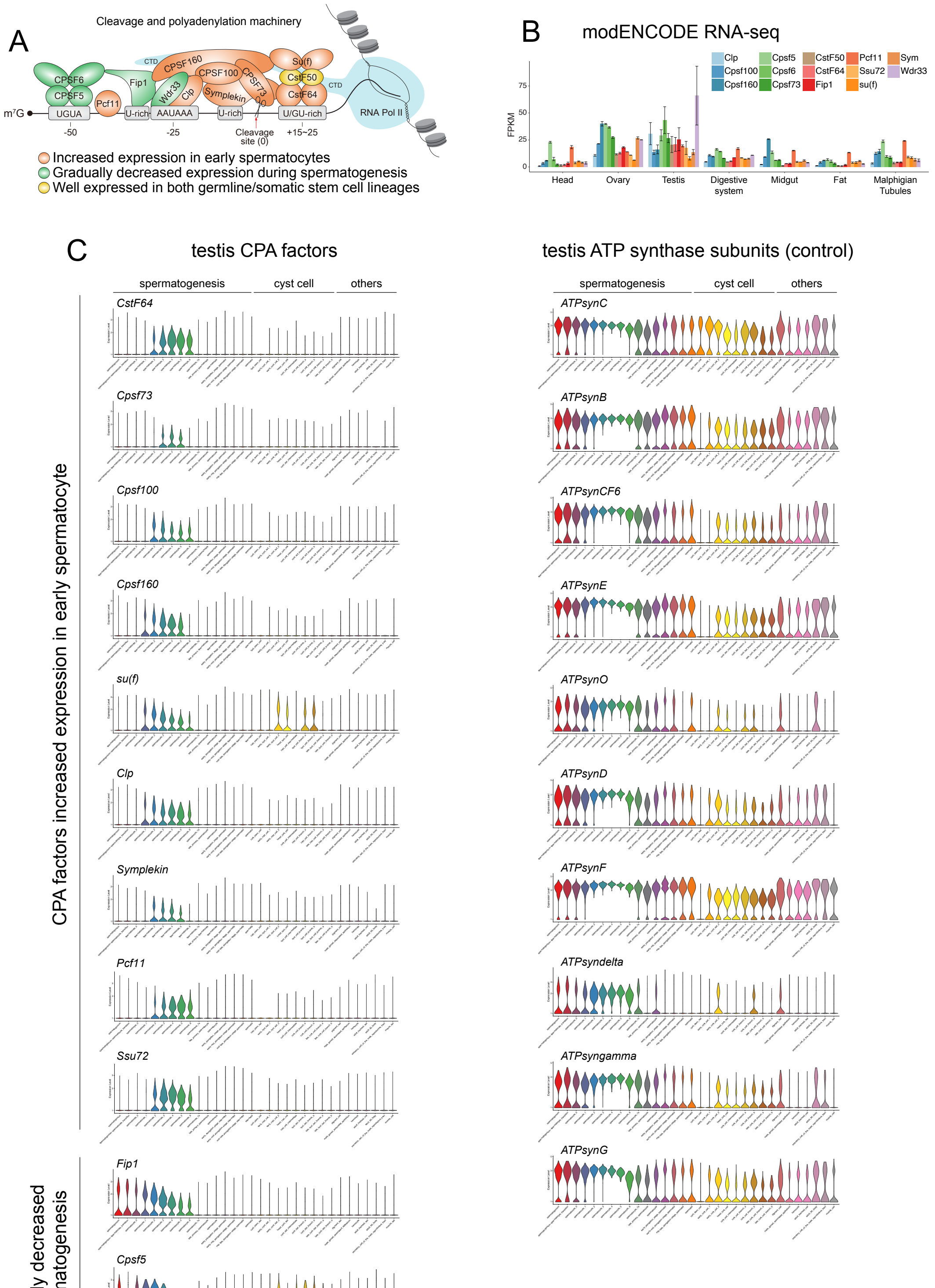

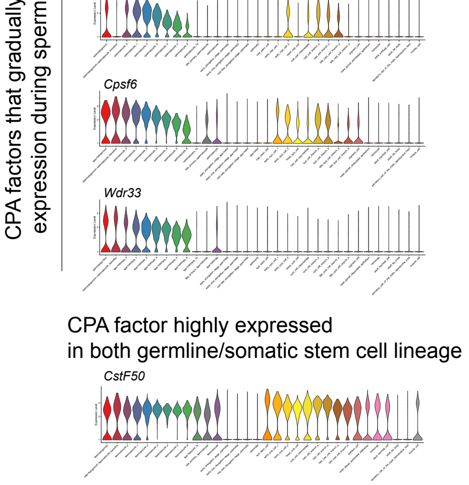

Supplementary Figure 8. Most cleavage and polyadenylation (CPA) factors are coordinately induced in early spermatocytes.

(A) Summary of core cleavage and polyadenylation (CPA) machinery and their overall expression trends in testis. (B) Expression of CPA factors in bulk tissue datasets from the modENCODE project shows global upregulation in gonads. Data are presented as mean values +/− SD pooled from three independent experiments. (C) Violin plots indicating the expression of the indicated factors in FCA testis data. Celltypes are arranged in order of the germline stem cell lineage for spermatogenesis, a somatic cyst stem cell lineage, and other somatic cell types that comprise the testis. (Left) Expression of CPA factors is generally low outside of the germline, except for *Cpsf6* and *su(f)* which have some expression in the cyst lineage. Otherwise, all CPA factors are generally expressed at highest levels in spermatocytes, although some of them have substantial expression in spermatogonia (stem cells, i.e. *Cpsf6*, *Wdr33* and *Fip1*). (Right) For comparison, the expression of ATP synthase subunits is shown across testis cell types, indicating that members of this housekeeping complex can be reliably detected

Supplementary Figure 8
Lee et al

Supplementary Table 1. RT-PCR primer sequences used in this study

Name	Sequence (5' -> 3')
mei-P26-Primer1-F	CGTGTGCCCTCCTTTAATGT
mei-P26-Primer1-R	CGCATTCCTACATCCGTTTT
mei-P26-Primer2-F	ACTTAGGCCCCAAAGCAAAT
mei-P26-Primer2-R	TGCGAAAATGGAAAGGAAAG
mei-P26-Primer3-F	TCCGAGGGCTATGTGGTTAC
mei-P26-Primer3-R	CGACTGATGGACGATTGTTG
ssg-Primer1-F	CCTATGACAACCACCCCAGT
ssg-Primer1-R	GGTTTTGCTTTTGCTTTTGC
ssg-Primer2-F	GAATGGAGAAAGCCAATCCA
ssg-Primer2-R	TTTTGAAATTGCGGTTGATG
ssg-Primer3-F	CGAGCATCGTTCCTCTATCC
ssg-Primer3-R	TGCTTCTTCTACGGCTTCCT
Oaf-short-F	TTCGCATCACCAGTCATCAT
Oaf-short-R	AGCGGAAAAAGTGTCAGCAT
Oaf-long-F	CATTTGGGGAATGGTGAATG
Oaf-long-R	AAACAGCCATTTTCGAGTGG
Lig-short-F	GCACACCCGCCAATACATTG
Lig-short-R	ACATGCGGTTCAACAAATCA
Lig-long-F	CGCGAACGACTTTTATAGCC
Lig-long-R	CGCTGCTCTTGCTGTAGATG
Bbc-short-F	ATTCACCGTCGATGTTTTGG
Bbc-short-R	CTGTCTCCCAGCAGCCTTAG
Bbc-long-F	AGCGCACTACCTACCTTGGA
Bbc-long-R	CCGTGCGAGCTACAAACATA
