# Supplementary material for: Diverse cell-specific patterns of alternative polyadenylation in Drosophila
Source: Nat Commun. 2022 Sep 13;13:5372. doi: 10.1038/s41467-022-32305-0 (PMC9470587; doi:10.1038/s41467-022-32305-0)
Supplement: Supplementary file 2 — Description of Additional Supplementary Files [file 41467_2022_32305_MOESM2_ESM.pdf]

Description of Additional Supplementary Files

File Name: Supplementary Data 1
Description: Complete analysis relative 3' isoform usage of signature APA genes across all cell types in the Fly Cell Atlas.

File Name: Supplementary Data 2
Description: Numbers of cells used for each of the Fly Cell Atlas cell type assignments.

File Name: Supplementary Data 3
Description: Genes expressing distinctive TUTR-APA or ALE-APA isoforms in neurons.

File Name: Supplementary Data 4
Description: Genes undergoing TUTR-APA or ALE-APA shifts in the male germline lineage.

File Name: Supplementary Data 5
Description: Genes undergoing TUTR-APA or ALE-APA shifts in the intestinal stem cell lineage.

File Name: Supplementary Data 6
Description: List of RNA binding proteins (RBPs) assessed in this study.

File Name: Supplementary Data 7
Description: RBPs with differential cell-specific expression within cell types of the FCA body, gut and testis datasets.

File Name: Supplementary Data 8
Description: RBPs with differential cell-specific expression between male germline stem cells and spermatocytes.

File Name: Supplementary Data 9
Description: Relative numbers of miRNA binding sites discovered from 3' UTRs of spermatogenesis shortening genes.

File Name: Supplementary Data 10
Description: Relative numbers of RBP binding sites discovered from 3' UTRs of spermatogenesis shortening genes.
