# Supplementary material for: Diverse cell-specific patterns of alternative polyadenylation in Drosophila
Source: Nat Commun. 2022 Sep 13;13:5372. doi: 10.1038/s41467-022-32305-0 (PMC9470587; doi:10.1038/s41467-022-32305-0)
Supplement: Supplementary file 13 — Reporting Summary [file 41467_2022_32305_MOESM13_ESM.pdf]

nature portfolio

Corresponding author(s): Eric C. Lai

Last updated by author(s): Jun 22, 2022

Reporting Summary

Nature Portfolio wishes to improve the reproducibility of the work that we publish. This form provides structure for consistency and transparency in reporting. For further information on Nature Portfolio policies, see our Editorial Policies and the Editorial Policy Checklist.

Statistics

For all statistical analyses, confirm that the following items are present in the figure legend, table legend, main text, or Methods section.

n/a	Confirmed	
☐	☒	The exact sample size (*n*) for each experimental group/condition, given as a discrete number and unit of measurement
☐	☒	A statement on whether measurements were taken from distinct samples or whether the same sample was measured repeatedly
☐	☒	The statistical test(s) used AND whether they are one- or two-sided<br>*Only common tests should be described solely by name; describe more complex techniques in the Methods section.*
☐	☒	A description of all covariates tested
☐	☒	A description of any assumptions or corrections, such as tests of normality and adjustment for multiple comparisons
☐	☒	A full description of the statistical parameters including central tendency (e.g. means) or other basic estimates (e.g. regression coefficient) AND variation (e.g. standard deviation) or associated estimates of uncertainty (e.g. confidence intervals)
☐	☒	For null hypothesis testing, the test statistic (e.g. *F*, *t*, *r*) with confidence intervals, effect sizes, degrees of freedom and *P* value noted<br>*Give P values as exact values whenever suitable.*
☒	☐	For Bayesian analysis, information on the choice of priors and Markov chain Monte Carlo settings
☒	☐	For hierarchical and complex designs, identification of the appropriate level for tests and full reporting of outcomes
☒	☐	Estimates of effect sizes (e.g. Cohen's *d*, Pearson's *r*), indicating how they were calculated

Our web collection on statistics for biologists contains articles on many of the points above.

Software and code

Policy information about availability of computer code

Data collection	The following tools were used in this study: R 4.2.0, Seurat 4.0.1, Python 3.6, samtools 1.15, bedtools 2.30.0, cellranger 6.1.2, and LABRAT (https://github.com/TaliaferroLab/LABRAT)
Data analysis	In-house computational codes that were implemented to reproduce the main results in this study are available on GitHub at the following link: https://github.com/imsjay/scAPA_FCA_paper

For manuscripts utilizing custom algorithms or software that are central to the research but not yet described in published literature, software must be made available to editors and reviewers. We strongly encourage code deposition in a community repository (e.g. GitHub). See the Nature Portfolio guidelines for submitting code & software for further information.

Data

Policy information about availability of data

All manuscripts must include a data availability statement. This statement should provide the following information, where applicable:

- Accession codes, unique identifiers, or web links for publicly available datasets
- A description of any restrictions on data availability
- For clinical datasets or third party data, please ensure that the statement adheres to our policy

The raw sequencing data and the cell type annotation information generated from the Fly Cell Atlas (FCA) project was used in this study. The 10X Genomics raw data is available at https://www.ebi.ac.uk/arrayexpress/experiments/E-MTAB-10519/. H5AD files for each correspond fly tissues are available at https://flycellatlas.org/.

Field-specific reporting

Please select the one below that is the best fit for your research. If you are not sure, read the appropriate sections before making your selection.

- [x] Life sciences
- [ ] Behavioural & social sciences
- [ ] Ecological, evolutionary & environmental sciences

For a reference copy of the document with all sections, see nature.com/documents/nr-reporting-summary-flat.pdf

Life sciences study design

All studies must disclose on these points even when the disclosure is negative.

Sample size	For the scRNA-seq data analysis, the sample size was chosen by cell numbers, especially sample size for 3'UTR quantification analysis was chosen based upon the number of reads satisfying the threshold described in the method section. The rt-qPCR experiment in Figure 1 and Figure 4 was performed with two biological independent samples.
Data exclusions	Outliers to select data for the analysis was described in the method section. Except for this, all data sets were included in the paper and used for the downstream analysis.
Replication	N/A, There is a single assignment of individual cell types in the Fly Cell Atlas data.
Randomization	N/A, There is a single assignment of individual cell types in the Fly Cell Atlas data.
Blinding	The investigators were blinded during data processing and analyses.

Reporting for specific materials, systems and methods

We require information from authors about some types of materials, experimental systems and methods used in many studies. Here, indicate whether each material, system or method listed is relevant to your study. If you are not sure if a list item applies to your research, read the appropriate section before selecting a response.

Materials & experimental systems

n/a	Involved in the study	
x		Antibodies
x		Eukaryotic cell lines
x		Palaeontology and archaeology
x		Animals and other organisms
x		Human research participants
x		Clinical data
x		Dual use research of concern

Methods

n/a	Involved in the study	
x		ChIP-seq
x		Flow cytometry
x		MRI-based neuroimaging
